# Lymphocyte‐Activation Gene 3 Facilitates Pathological Tau Neuron‐to‐Neuron Transmission

**DOI:** 10.1002/advs.202303775

**Published:** 2024-02-07

**Authors:** Chan Chen, Ramhari Kumbhar, Hu Wang, Xiuli Yang, Kundlik Gadhave, Cyrus Rastegar, Yasuyoshi Kimura, Adam Behensky, Sumasri Kotha, Grace Kuo, Sruthi Katakam, Deok Jeong, Liang Wang, Anthony Wang, Rong Chen, Shu Zhang, Lingtao Jin, Creg J. Workman, Dario A. A. Vignali, Olga Pletinkova, Hongpeng Jia, Weiyi Peng, David W. Nauen, Philip C. Wong, Javier Redding‐Ochoa, Juan C. Troncoso, Mingyao Ying, Valina L. Dawson, Ted M. Dawson, Xiaobo Mao

**Affiliations:** ^1^ Neuroregeneration and Stem Cell Programs Institute for Cell Engineering Johns Hopkins University School of Medicine Baltimore MD 21205 USA; ^2^ Department of Neurology Johns Hopkins University School of Medicine Baltimore MD 21205 USA; ^3^ Department of Molecular Medicine University of Texas Health Science Center at San Antonio San Antonio TX 78229 USA; ^4^ Department of Immunology University of Pittsburgh School of Medicine Pittsburgh PA 15213 USA; ^5^ Tumor Microenvironment Center UPMC Hillman Cancer Center Pittsburgh PA 15213 USA; ^6^ Cancer Immunology and Immunotherapy Program UPMC Hillman Cancer Center Pittsburgh PA 15213 USA; ^7^ Department of Pathology Division of Neuropathology Johns Hopkins University School of Medicine Baltimore MD 21205 USA; ^8^ Division of Pediatric Surgery Department of Surgery Johns Hopkins University School of Medicine Baltimore MD 21205 USA; ^9^ Department of Biology and Biochemistry University of Houston 3517 Cullen Blvd. Houston TX 77204 USA; ^10^ Hugo W. Moser Research Institute at Kennedy Krieger 707 North Broadway Baltimore MD 21205 USA; ^11^ Adrienne Helis Malvin Medical Research Foundation New Orleans LA 70130–2685 USA; ^12^ Department of Physiology Johns Hopkins University School of Medicine Baltimore MD 21205 USA; ^13^ Solomon H. Snyder Department of Neuroscience Johns Hopkins University School of Medicine Baltimore MD 21205 USA; ^14^ Department of Pharmacology and Molecular Sciences Johns Hopkins University School of Medicine Baltimore MD 21205 USA; ^15^ Institute for NanoBioTechnology Johns Hopkins University Baltimore MD 21218 USA; ^16^ Department of Materials Science and Engineering Johns Hopkins University Baltimore MD 21218 USA; ^17^ Present address: Department of Pathology and Anatomical Sciences Jacobs School of Medicine and Biomedical Sciences University at Buffalo Buffalo NY 14260 USA; ^18^ Present address: Department of Anesthesiology, West China Hospital, Sichuan University The Research Units of West China (2018RU012)‐Chinese Academy of Medical Sciences, West China Hospital, Sichuan University

**Keywords:** lymphocyte‐activation gene 3, cell‐to‐cell transmission, receptor, Tau, Tau preformed fibrils

## Abstract

The spread of prion‐like protein aggregates is a common driver of pathogenesis in various neurodegenerative diseases, including Alzheimer's disease (AD) and related Tauopathies. Tau pathologies exhibit a clear progressive spreading pattern that correlates with disease severity. Clinical observation combined with complementary experimental studies has shown that Tau preformed fibrils (PFF) are prion‐like seeds that propagate pathology by entering cells and templating misfolding and aggregation of endogenous Tau. While several cell surface receptors of Tau are known, they are not specific to the fibrillar form of Tau. Moreover, the underlying cellular mechanisms of Tau PFF spreading remain poorly understood. Here, it is shown that the lymphocyte‐activation gene 3 (Lag3) is a cell surface receptor that binds to PFF but not the monomer of Tau. Deletion of Lag3 or inhibition of Lag3 in primary cortical neurons significantly reduces the internalization of Tau PFF and subsequent Tau propagation and neuron‐to‐neuron transmission. Propagation of Tau pathology and behavioral deficits induced by injection of Tau PFF in the hippocampus and overlying cortex are attenuated in mice lacking Lag3 selectively in neurons. These results identify neuronal Lag3 as a receptor of pathologic Tau in the brain，and for AD and related Tauopathies, a therapeutic target.

## Graphical abstract

Tau pathology spreads progressively in a hierarchical pattern linked to cognitive decline. Human and experimental studies show Alzheimer's and related Tauopathies progress via misfolded Tau propagating between cells. Lag3 is identified as a receptor enabling Tau fibril cell–cell transmission. This significantly advances the understanding of Tau transmission mechanisms and presents Lag3‐blocking therapy as a novel approach.

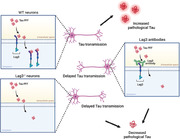

## 1 Significance

Tau pathology exhibits a clear progressive and hierarchical spreading pattern, which correlates with cognitive impairment. Human postmortem and experimental studies have shown that disease progression of Alzheimer's disease (AD) and related Tauopathies is driven by pathogenic tau, which can propagate Tau pathology by entering cells and templating misfolding and aggregation. However, the underlying cellular mechanisms of pathogenic Tau fibrils spreading remain poorly understood. Here, we identified Lag3 as a pathogenic fibrils specific cell surface receptor. Our results represent a major advance toward understanding the molecular mechanisms of cell‐to‐cell transmission of pathologic Tau fibrils in AD and related Tauopathies. Our study provides a novel understanding of the internalization of pathogenic Tau fibrils and provides innovative insight into Lag3‐related therapy.

## 2 Introduction

In Alzheimer's disease (AD), the burden of Tau pathology is highly correlated with the severity of cognitive decline.^[^ ^1^ ^]^ Emerging postmortem and experimental studies indicate that pathogenic Tau spreads in a stereotypical manner, driving disease progression.^[^ ^2^ ^]^ Pathogenic Tau preformed fibrils (PFF) serve as prion‐like seeds that induce the misfolding of endogenous tau monomer, resulting in cell‐to‐cell propagation in Tauopathies.^[^ ^2c,d,f^ ^]^ Recent work has identified several receptors of Tau uptake, including heparan sulfate proteoglycans (HSPGs)^[^ ^3^ ^]^ and low‐density lipoprotein receptor‐related protein 1 (LRP1).^[^ ^4^ ^]^ However, these identified receptors are not specific to Tau aggregates or other Tau species (e.g., monomer and soluble Tau).^[^ ^3^, ^4^ ^]^ Of note, a substantial amount of Tau PFF still accumulates in LRP1 knocked‐down cells,^[^ ^4^ ^]^ which suggests that some other receptors or pathways could mediate the endocytosis of Tau PFF. Identification of a Tau‐fibril–specific receptor could provide the opportunity to validate its role in initiating Tauopathy and offer a potential therapeutic target for AD and related Tauopathies.

While several Tau receptors (e.g., HSPGs, LRP1) have shown to bind α‐synuclein (α‐syn),^[^ ^3^, ^5^ ^]^ we identified lymphocyte‐activation gene 3 (Lag3) as a major cell‐surface receptor that specifically binds with α‐syn fibrils, but not to the α‐syn monomer.^[^ ^6^ ^]^ Depletion of Lag3 inhibits the uptake and reduces the subsequent α‐syn pathology.^[^ ^6^, ^7^ ^]^ To determine whether Lag3 is specific for α‐syn fibrils but not to other prion‐like proteins, we further tested the interaction between Lag3 and tau PFF. The results showed no interaction between Lag3 and heparin‐induced Tau (hep‐Tau) PFF.^[^ ^6^ ^]^ A recent study showed that hep‐Tau PFF is not an active seed in vivo, as compared to self‐seeded tau or the AD brain extracts‐induced Tau (AD‐Tau) PFF.^[^ ^2d^ ^]^ Thus, we wondered whether bioactive tau, but not hep‐tau, fibrils bind to Lag3. Here, we show that Lag3 is a cell surface receptor specific for pathogenic fibrils but not the monomer of Tau. We employed genetic and biochemical approaches to test the requirement of Lag3 for pathogenic Tau propagation and neuron‐to‐neuron transmission.

### 2.1 Preparation and Characterization of Tau PFF

Intracerebral inoculation of Tau PFF purified from AD brains (AD‐Tau) resulted in robust Tau inclusions in anatomically connected brain regions in non‐transgenic mice.^[^ ^2d,f^ ^]^ We extracted AD‐Tau, seeded recombinant Tau (5/95%) for fibrillization, and sonicated to obtain Tau PFF as published.^[^ ^2d^ ^]^ We characterized the Tau‐PFF with transmission electron microscopy (TEM) (Figure S1a,b, Supporting Information) and thioflavin T (ThT) assay (Figure S1c,d, Supporting Information).

We labeled Tau with biotin to distinguish it from endogenous Tau, and then investigated the interaction between Tau‐biotin species and primary cortical neurons using a cell surface binding assay. Tau‐biotin PFF binds to primary cortical neurons as detected with streptavidin‐alkaline phosphatase (AP) staining^[^ ^8^ ^]^ (Figure S2a,b, Supporting Information). Tau‐biotin PFF binding to neurons is saturable with an apparent dissociation constant (KD) of 276 nm, whereas both hep‐Tau–biotin PFF and Tau‐biotin monomer fail to show the saturable binding (KD > 3000 nm) (Figure S2a,b, Supporting Information). Biotin as the negative control showed a minimal binding signal (Figure S2a,b, Supporting Information), suggesting the existence of PFF‐specific receptor(s) and/or binding site(s) for Tau PFF. These results further suggest that heparin can reduce the interaction between neuronal surface and Tau‐biotin PFF.

### 2.2 The Interaction between Lag3 and Tau‐Biotin PFF

**Figure 1:**
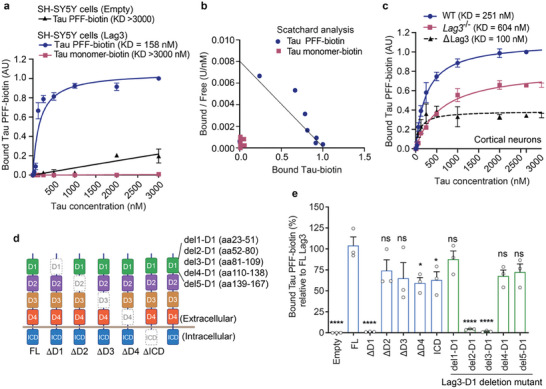
Lag3 binds with Tau PFF, but not Tau monomer. a) Tau‐biotin PFF binds to SH‐SY5Y cells transiently transfected with Lag3 in a saturable manner, as a function of Tau‐biotin total concentration (monomer equivalent for PFF preparations) while SH‐SY5Y cells that are transfected with empty vector exhibit Tau‐biotin PFF binding at high concentrations. b) Scatchard analysis. Data are the means ± SEM, *n* = 3 independent experiments. c) The binding of Tau‐biotin PFF to mouse primary cortical neurons is reduced by Lag3 knockout (Lag3^−/−^). Tau‐biotin PFF WT‐KD = 251 nm, Lag3^−/−^‐KD = 604 nm, estimated KD for neuronal Lag3 (dotted line, ΔLag3 = wild type minus Lag3^−/−^) is 100 nm. Data are the means ± SEM, *n* = 3 independent experiments. d) Schematics of deletion mutants of Lag3 ectodomains. e) Tau‐biotin PFF binding to Lag3 and deletion mutants of Lag3: extracellular domains (ΔD1–ΔD4), intracellular domain (ΔICD, and subdomains of D1 domain (del1—5‐D1) (*n* = 3). ****P < 0.05, ****P < 0.0001 compared to FL, n.s., not significant, one‐way ANOVA followed by Dunnett's correction. Data are the means ± SEM.

In the well‐established cell surface binding assay,^[^ ^8^ ^]^ Tau‐biotin PFF at the indicated concentrations were administered individually into the Lag3‐expressing SH‐SY5Y cells, and the binding signals were detected with streptavidin‐AP and its substrate staining. Our results showed that Tau‐biotin PFF binds to Lag3 in a saturable manner, with a KD = 158 nm, whereas no signal was observed with the Tau‐biotin monomer (**Figure** **1**a,b and S3a,b, Supporting Information).

We have determined that Lag3 is expressed in neurons using two in vivo systems.^[^ ^7b^ ^]^ Thus, we further studied the interaction between Lag3 and pathogenic Tau in neurons. WT mouse cortical neurons demonstrated Tau‐biotin PFF‐binding (KD = 251 nm), whereas Lag3^−/−^ mouse cortical neurons had reduced Tau‐biotin PFF‐binding (Figure 1c and Figure S3c,d, Supporting Information).

Specific binding of Tau‐biotin PFF to Lag3 in primary cortical neurons was determined by subtracting the Tau‐biotin PFF binding to Lag3^−/−^ neurons from the binding to WT neuron cultures, and reveals a KD of 100 nm (Figure 1c). Moreover, we substantiated the direct binding of Tau‐biotin PFF to human recombinant LAG3 with a KD of 3 nm using enzyme‐linked immunosorbent assay (Figure S3e, Supporting Information).

Additionally, we employed the sucrose gradient method to prepare globular Tau oligomers in a 30% sucrose solution^[^ ^9^ ^]^ (Figure S4a,b, Supporting Information). Subsequent labeling of these Tau oligomers with biotin allowed us to conduct cell surface binding assays. Our findings revealed that Tau oligomers exhibited binding to Lag3‐expressing SH‐SY5Y cells with a KD of 1300 nm (Figure S4c,d, Supporting Information), indicating a comparatively weaker binding of Lag3 to Tau oligomers. Collectively, these results demonstrate the preferential binding of Lag3 to Tau‐biotin PFF, as opposed to Tau‐biotin monomers or oligomers.

Heparin can inhibit cellular uptake of Tau^[^ ^3a^ ^]^ by mimicking HSPGs binding with Tau. We further performed the cell surface binding assay to compare hep‐Tau–biotin PFF and AD‐Tau–induced Tau‐biotin PFF. The result is consistent with the previous work^[^ ^6a^ ^]^ that hep‐Tau–biotin PFF exhibited significantly less binding to Lag3‐transfected SH‐SY5Y cells (Figure S5a,b, Supporting Information), compared to AD‐Tau–induced Tau PFF‐biotin. Moreover, when we pretreated primary cortical neurons (Figure S6a,b, Supporting Information) and SH‐SY5Y cells expressing Lag3 with heparin (Figure S6c,d, Supporting Information), we observed reduced Tau‐biotin PFF binding to the cell surface.

Additionally, to investigate the Tau‐biotin PFF binding to Lag3 in a Tau conformation‐dependent manner, we extracted Tau seeds from brain samples of patients with neuropathologically confirmed progressive supranuclear palsy (PSP) and Pick's disease (PiD, which are subtypes under the fronto‐temporal lobar degeneration group of disorders). Subsequently, we generated Tau PFF by allowing the tau monomer to undergo a 7 days aggregation reaction with 10% patient‐isolated Tau seeds^[^ ^2d^ ^]^ (Figure S7a, Supporting Information).

The Tau PFF derived from PSP exhibited a binding affinity to Lag3 with a KD of 227 nm, while Tau PFF from PiD demonstrated a KD of 271 (Figure S7b–d, Supporting Information). This indicates varying binding affinities between Tau PFF from distinct Tauopathies and Lag3. Furthermore, we conducted binding assays with amyloid‐β (Aβ) PFF on Lag3‐expressing SH‐SY5Y cells to explore interactions with other fibril structures. In the setting of low Aβ PFF binding to empty vector transfected SH‐SY5Y cells Aβ PFF binds to Lag3 with a KD of 310 nm (Figure S8, Supporting Information).

Lag3 has an ectodomain composed of four immunoglobulin (Ig)‐like domains (D1–D4).^[^ ^10^ ^]^ To determine the Tau‐biotin PFF‐binding domain, we sequentially deleted each Ig‐like domain of Lag3 and performed the cell surface binding assay with overexpression of Lag3 deletion mutants. These experiments revealed that Tau‐biotin PFF preferentially binds to the D1 domain, and deletion of the D2, D3, D4 or intracellular domain (ICD) reduced the binding to some extent (Figure 1d,e and Figure S9a,b, Supporting Information). As deletion of the D1 can eliminate the Tau‐biotin PFF‐binding to Lag3, additional deletions of D1 subdomains (del1—5) were examined. Del2‐D1 and Del3‐D1 can abolish the Tau‐biotin PFF‐binding to Lag3, whereas del1‐D1, del4‐D1, and del5‐D1 did not show significantly reduced binding to Lag3 (Figure 1e and Figure S9a,c, Supporting Information). These results show that residues 52–109 in the D1 domain are essential for the Lag3 interaction with Tau‐biotin PFF.

### 2.3 Neurons Uptake Tau PFF via Lag3

**Figure 2:**
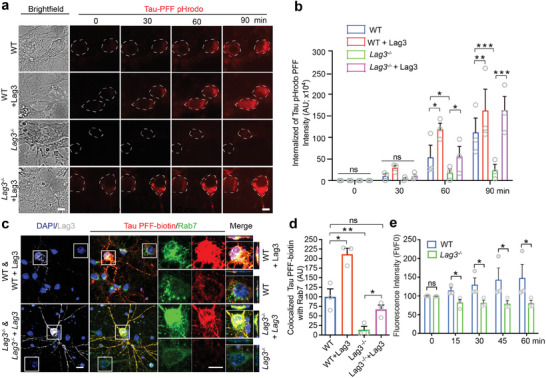
Lag3 is required for neuronal uptake of Tau PFF. a) Live image analysis of the endocytosis of Tau PFF‐pHrodo in WT, WT+Lag3, Lag3^−/−^, and Lag3^−/−^+Lag3 neurons at indicated time points. Increased red fluorescence indicates higher endocytosis. Scale bar, 10 µm. b) Quantification of Tau PFF‐pHrodo, *n* = 3 independent experiments. c) Lag3^−/−^ reduced the neuronal uptake of Tau‐biotin PFF co‐localized with endosome marker (Rab7). WT or Lag3^−/−^ neurons were cultured, and received transient transfection with Lag3. WT, WT+Lag3, Lag3^−/−^, and Lag3^−/−^+Lag3 neurons were treated with 500 nm Tau‐biotin PFF for 2 h. Scale bar, 10 µm. d) Quantification of (c) from three independent experiments. One‐way analysis of variance (ANOVA) with Tukey's correction. e) Quantification of time series imaging of calcium‐dependent fluorescence of WT and Lag3^−/−^ neurons. Neurons were treated with 1 εm Fluo‐2 acetoxymethyl (AM) ester for 30 min followed by 500 nm Tau PFF and live images were acquired for the indicated time period. Error bars represent SEM. *P < 0.05, **P < 0.01, ***P < 0.001.

To determine whether Lag3 is involved in the endocytosis of Tau PFF, pHrodo red dye was conjugated with Tau PFF, so that Tau‐pHrodo PFF can fluoresce as pH decreases from the neutral cytosolic pH to the acidic pH of the endosome and lysosome.^[^ ^11^ ^]^ Tau‐pHrodo PFF fluoresced and the fluorescent signal was increased in WT cortical neurons with time via live imaging, whereas Lag3^−/−^ neurons exhibited minimal fluorescent signal (**Figure** **2**a,b). Overexpression of Lag3 by lentiviral transduction in WT neurons enhanced the fluorescent signal, and overexpression of Lag3 in Lag3^−/−^ neurons restored the uptake of Tau‐pHrodo PFF (Figure 2a,b). These results show that neuronal Lag3 can uptake Tau‐pHrodo PFF.

Rab7 is a small guanosine triphosphatase that belongs to the Rab family and controls transport to the late endosome and lysosomes.^[^ ^12^ ^]^ We used Rab7 as an endocytosis marker to assess the co‐localized signal with Tau‐biotin PFF as an additional monitor of uptake. We found that Tau‐biotin PFF was co‐localized with Rab7 in WT cortical neurons, and that there was a significantly reduced co‐localized signal in Lag3^−/−^ cortical neurons (Figure 2c,d). Overexpression of Lag3 in WT and Lag3^−/−^ neurons enhanced the intensity of Tau‐biotin PFF co‐localizing with Rab7 (Figure 2c,d).

Given the relevance of calcium in Tau pathogenesis^[^ ^13^ ^]^ and considering our previous demonstration that Lag3 knockout neurons exhibit increased calcium influx upon α‐synuclein PFF treatment,^[^ ^6a^ ^]^ we monitored calcium influx in response to Tau PFF. Perfusion of Tau PFF (500 nm) onto WT cortical neurons caused a gradual increase in intracellular calcium levels (Figure 2e and Figure S10, Supporting Information). In contrast, a significant reduction of Tau PFF‐induced calcium influx was observed in Lag3^−/−^ cortical neurons (Figure S10, Supporting Information).

Taken together, these data indicate that after Tau PFF administration, Lag3 is required for the neuronal uptake of Tau PFF, and deletion of Lag3 can reduce the subsequent calcium influx induced by Tau PFF.

### 2.4 Deletion of Lag3 Attenuates Tau PFF‐Induced Pathology

**Figure 3:**
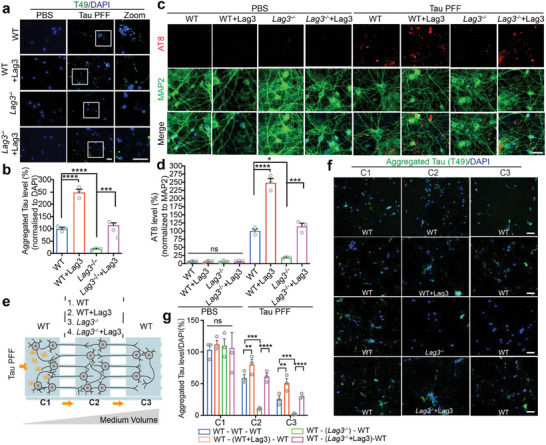
Lag3 is required for Tau pathology propagation and neuron‐to‐neuron transmission. a) Depletion of Lag3 reduces abnormal Tau aggregation (T49). WT, WT+Lag3, Lag3^−/−^, and Lag3^−/−^+Lag3 primary cortical neurons at 7 DIV were treated with Tau PFF or PBS for 14 days. Neurons were fixed with methanol to remove soluble Tau as described^[^ ^2d^ ^]^ and immunostained with T49, a mouse Tau‐specific monoclonal antibody. Abnormal Tau aggregates were assessed by immunostaining with T49 antibody. Scale bar, 20 µm. b) Quantification of (a), *n* = 3 independent experiments. Aggregates Tau levels were normalized with DAPI signal. Error bars represent SEM. Statistical significance was determined using one‐way ANOVA followed with Tukey's correction, c) Depletion of Lag3 reduced hyper‐phosphorylated Tau at Ser202, Thr205 (AT8). Scale bar, 20 µm. d) Quantification of panel (c), *n* = 3 independent experiments. AT8 intensities were normalized with neuronal marker MAP2. Error bars represent SEM. Statistical significance was determined using one‐way ANOVA followed with Tukey's correction. e) Schematic of microfluidic neuron device with three chambers (chamber 1‐2‐3, C1‐C2‐C3), to separate neuron cultures. f) Transmission of aggregated Tau, 14 days after the administration of Tau PFF in C1. Different neurons in C2, listed as C1‐(C2)‐C3, are: WT‐(WT)‐WT, WT‐(WT+Lag3)‐WT, WT‐(Lag3^−/−^)‐WT, WT‐(Lag3^−/−^+Lag3)‐WT. Scale bar, 100 µm. g) Quantification of (f); error bar represents SEM, *n* = 3 independent experiments. Aggregates Tau levels were normalized with DAPI signal. Statistical significance was determined using one‐way ANOVA followed by Tukey's correction, *P < 0.01, **P < 0.01, ***P < 0.001, ****P < 0.0001, n.s., not significant.

To determine the role of Lag3 in mediating Tau pathology, we administered Tau PFF in WT and Lag3^−/−^ primary cortical neurons on 7 days in vitro (DIV) and incubated for a further 14 days, and then assessed the Tau pathology, including the insoluble Tau and hyper‐phosphorylated Tau (p‐Tau). We fixed the neurons with methanol to remove soluble Tau as previously published^[^ ^2d^ ^]^ and immunostained with T49, a mouse Tau‐specific monoclonal antibody. The results show a significant increase of insoluble Tau in WT neurons treated with Tau PFF, compared to PBS‐treated neurons (**Figure** **3**a,b). Depletion of Lag3 significantly reduced the insoluble Tau (≈80%) compared to WT neurons (Figure 3a,b). Overexpression of Lag3 by lentiviral transduction resulted in a significant increase of insoluble Tau in WT and Lag3^−/−^ neurons (Figure 3a,b). Furthermore, we assessed the p‐Tau level with AT8 immunostaining using anti‐p‐Tau (Ser202, Thr205). The results show a substantial p‐Tau signal in WT neurons induced by Tau PFF as published.^[^ ^2d^ ^]^ In contrast, 75% of p‐Tau signal was decreased in Lag3^−/−^ neurons (Figure 3c,d). Overexpression of Lag3 significantly enhanced p‐Tau signal in WT neurons and restored p‐Tau signal in Lag3^−/−^ neurons (Figure 3c,d).

### 2.5 Neuron‐to‐Neuron Transmission of Tau Pathology Is Reduced in Lag3^−/−^ Neurons

To examine the neuron‐to‐neuron transmission of Tau pathology, we used a microfluidic device (three separated chambers connected in tandem by two series of microgrooves) as previously described^[^ ^6^, ^7^, ^14^ ^]^ (Figure 3e). Four experimental and control groups with primary cortical neurons were cultured in successive chambers (C1)‐(C2)‐(C3): (WT)‐(WT)‐(WT), (WT)‐(WT+Lag3)‐(WT), (WT)‐(Lag3^−/−^)‐(WT), and (WT)‐(Lag3^−/−^+Lag3)‐(WT). Using this system, the neuron‐to‐neuron transmission of Tau pathology was monitored via anti‐T49 (insoluble Tau). In (WT)‐(WT)‐(WT) cortical neuron cultures, Tau PFF was administered into C1, which led to Tau pathology transmission to C2 and later C3 to (Figure 3f). In the C1 of (WT)‐(Lag3^−/−^)‐(WT) cortical neuron cultures, there is no significant difference of Tau pathology compared to C1 of (WT)‐(WT)‐(WT), which indicates that the levels of pathogenic Tau from WT neurons (donor cells) were similar. Of note, a significant reduction of insoluble Tau can be observed in Lag3^−/−^ neurons in C2, which further prevented Tau propagation in WT neurons in C3 (Figure 3f,g). Overexpression of Lag3 by lentiviral‐transduction in C2 of (WT)‐(WT+Lag3)‐(WT) and (WT)‐(Lag3^−/−^+Lag3)‐(WT) can significantly increase the neuron‐to‐neuron transmission of Tau pathology in C2 and C3 (Figure 3f,g). These results show that Lag3 is required for the neuron‐to‐neuron transmission of Tau pathology.

### 2.6 Antibodies to Lag3 Block Neuronal Tau Propagation and Neuron‐to‐Neuron Transmission Induced by Tau PFF

**Figure 4:**
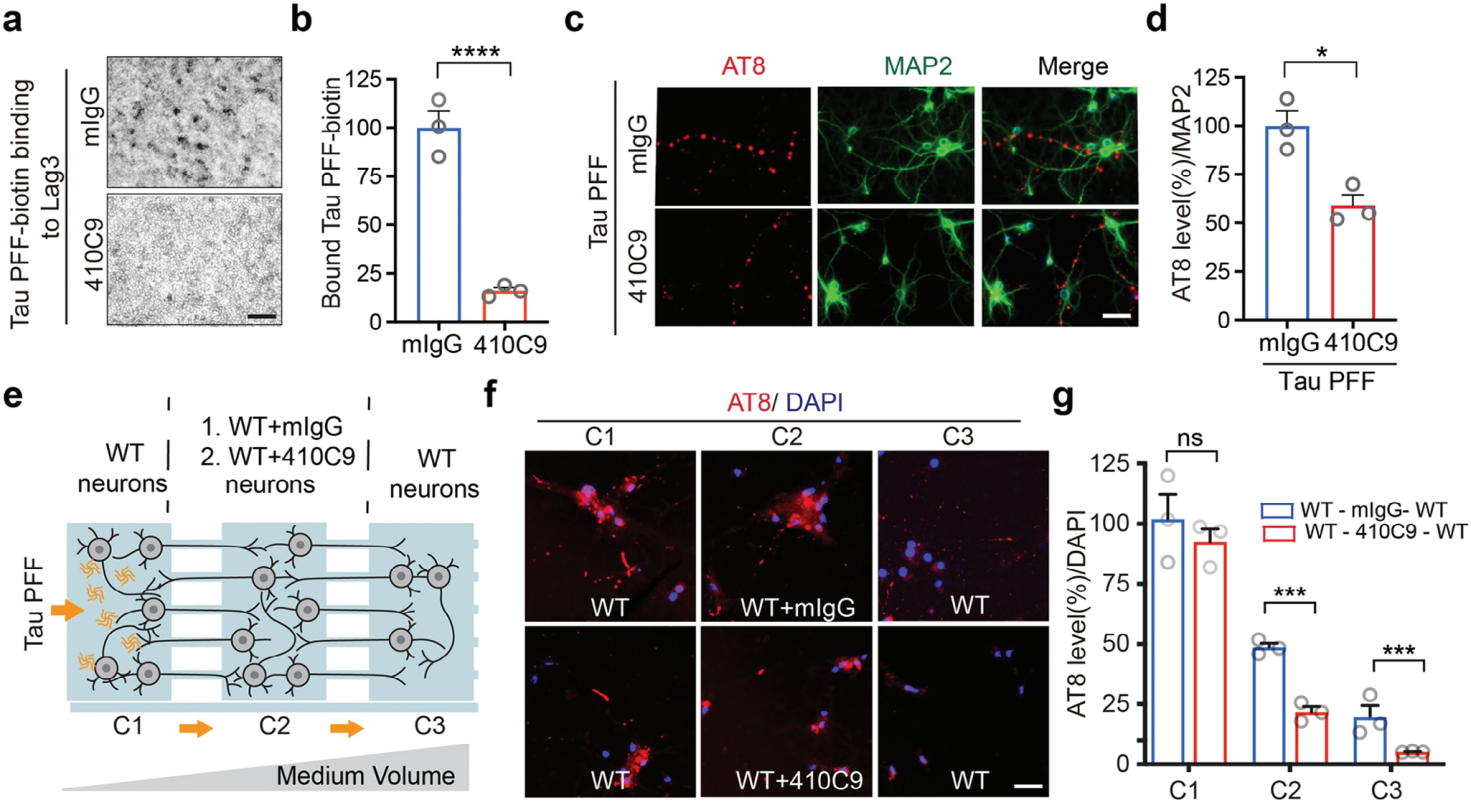
Lag3 antibodies block Tau PFF binding to Lag3, and subsequent pathologic propagation and neuron‐to‐neuron transmission. a) Anti‐Lag3 410C9 blocks the binding of Tau‐biotin PFF to Lag3‐expressing SH‐SY5Y cells. b) Quantification of (a); Error bars represent means ± SEM, *n* = three independent experiments, Student's *t*‐test. ****P < 0.0001. c) AT8 phosphorylated Tau (P‐Tau) was reduced by 410C9 (30 nm) in primary cortical neurons. d) Quantification of c) Error bars represent means ± SEM, *n* = 3 independent experiments, hyper‐phosphorylated Tau at Ser202, Thr205 (AT8) levels were normalized with neuronal marker MAP2 signal. Student's *t*‐test. *P < 0.05, Scale bar, 50 µm. e) Schematic of a microfluidic device experimental design. The different combinations of neurons in C2, listed as C1‐(C2)‐C3, are: WT‐(WT+mIgG)‐WT, WT‐(WT+410C9)‐WT. f) Transmission of Tau pathology 14 days after the administration of Tau PFF in C1. 410C9 significantly reduces the neuron‐to‐neuron transmission of Tau pathology (P‐Tau). g) Quantification of (f). Error bars represent means ± SEM, *n* = 3 independent experiments, hyper‐phosphorylated Tau at Ser202, Thr205 (AT8) levels were normalized with DAPI intensity. Student's *t*‐test. ***P < 0.001, n.s., not significant. Scale bar, 50 µm.

To determine whether Lag3 can serve as a therapeutic target, we administered Lag3 antibodies into primary cortical neurons. Lag3 antibody 410C9^[^ ^15^ ^]^ (30 nm) significantly blocked the binding of Tau‐biotin PFF (250 nm) to Lag3‐overexpressing SH‐SY5Y cells, compared to the control IgG (**Figure** **4**a,b). Similarly, results were likewise shown by other Lag3 antibodies C9B7W^[^ ^16^ ^]^ (30 nm) (Figure S11a,b, Supporting Information) and 17B4 (30 nm) (Figure S11c,d, Supporting Information) for human LAG3. Furthermore, 410C9 reduced the Tau PFF‐induced p‐Tau in WT cortical neurons, compared to the control IgG (Figure 4c,d), and C9B7W showed similar efficacy (Figure S11e,f, Supporting Information). In the in vitro assay of neuron‐to‐neuron transmission of Tau pathology, treatment of 410C9 antibody in C2 can significantly block Tau pathology transmission in C2 and C3 of WT cortical neurons, compared to control IgG treatment (Figure 4e–g). Thus, these Lag3 antibody studies support the notion that Lag3 is required for propagation and transmission of Tau pathology in vitro.

### 2.7 Deletion of Lag3 Reduces Tau Pathology Transmission and Alleviates Behavioral Deficits In Vivo

**Figure 5:**
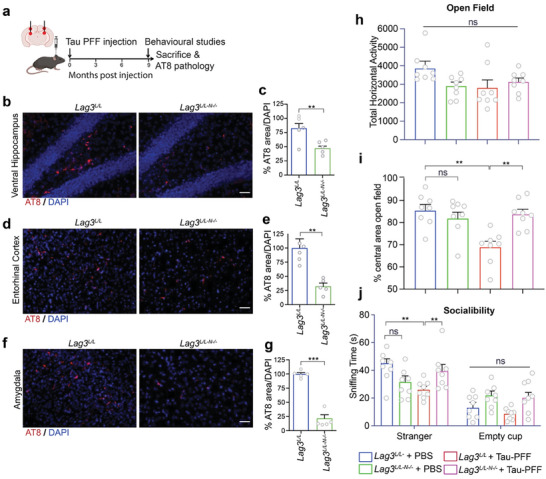
Tau pathologic spreading is reduced in Lag3 neuronal conditional knockout (Lag3^L/L‐N‐/−)^ mice and reduction of Tau PFF‐induced behavioral deficits. a) Schema of in vivo experiment. Stereotaxic injection of Tau PFF was performed into the dorsal hippocampus and overlying cortex in Lag3^L/L‐N‐/−^ mice and Lag3^L/L^ mice. Pathology and behavioral assessment were performed 9 months after Tau PFF injection. b–g) Immunostaining of P‐Tau in the ventral Hippocampus (b,c), entorhinal cortex (d,e), and amygdala (f,g) 9 months after Tau PFF injection. Error bars represent SEM, and *P*‐values were calculated with two‐tailed paired student *t*‐test. **P < 0.01, ***P < 0.001, *n* = 5 mice, Scale bar, 100 µm. h–j) Lag3^L/L‐N‐/−^ reduces the behavioral deficits in the open‐field test, total horizontal activity (h), % central area (i), and sniffing time in the social interaction test (j). Error bars represent SEM, and *P* values were calculated with one‐way ANOVA followed by Tukey's correction. n.s., not significant, **P < 0.01, ***P < 0.001.

To determine whether neuronal Lag3 is necessary for Tau pathology transmission in vivo, we bred Lag3^L/L‐YFP^ conditional knockout‐reporter mice^[^ ^17^ ^]^ (Lag3^L/L‐YFP^) with nestin^Cre^ mice (Jax Lab, strain: 003771)^[^ ^18^ ^]^ to generate the Lag3 neuronal conditional knockout (Lag3^L/L‐N‐/−^) mice. We stereotactically injected Tau PFF into the dorsal hippocampus of Lag3^L/L‐N‐/−^ and Lag3^L/L‐YFP^, with PBS serving as the control^[^ ^2d^ ^]^ (**Figure** **5**a). Nine months after the injection of Tau PFF, p‐Tau immunoreactivity was monitored in the ventral hippocampus (VH), entorhinal cortex (EC), and amygdala (Amy) (Figure 5b–g). Substantial p‐Tau signal can be found in Lag3^L/L‐YFP^ mice, while p‐Tau signal was significantly reduced in the VH (Figure 5b,c), EC (Figure 5d,e), and Amy (Figure 5f,g) of Lag3^L/L‐N‐/−^ mice. Our observations revealed a propagation pattern reminiscent of AD tauopathy. This typical spread is consistent with that described in prior studies evaluating spatiotemporal transmission of AD seeded tau^[^ ^2f^ ^]^. We observed tau aggregates spreading throughout the cortex and neocortical regions. Another pronounced region of Tau aggregates is evident proximal to the hippocampus, particularly within the fornix system, as shown in studies of seeded AD Tau pathology^[^ ^2f^ ^]^ (Figure S12a, Supporting Information).

To determine the role of neuronal Lag3 in mediating behavioral deficits induced by Tau PFF, we performed several behavioral tests as described.^[^ ^19^ ^]^ We assessed anxiety‐related behavior by analyzing time spent in the center zone versus the peripheral zone using the open field test.^[^ ^20^ ^]^ The results show that there is no significant difference in total horizontal activity in the Lag3^L/L‐YFP^ mice between the PBS and Tau PFF inoculated groups (Figure 5h), which is consistent with a prior study^[^ ^19^ ^]^, indicating that hippocampal injection of Tau PFF cannot cause motor dysfunction. We further assessed the staying time in the central area, which reflects anxiety. The results show that Tau PFF can significantly reduce the staying time in the central area in Lag3^L/L‐YFP^ mice compared to the PBS group (Figure 5i). In contrast, Lag3^L/L‐N‐/−^ mice alleviated the behavioral deficit in the Tau PFF group (Figure 5i). Further, we assessed cognitive impairment through sociability with the three‐chamber social interaction test. The results show that PBS treated mice exhibited normal sociability. In contrast, Tau PFF treated Lag3^L/L‐YFP^ mice exhibited significantly reduced sociability (Figure 5j and Figure S12b, Supporting Information). As expected, depletion of neuronal Lag3 significantly recovered social ability in the Tau PFF group, and there is no significant difference compared to PBS group (Figure 5j). To evaluate working memory, we tested Tau PFF injected Lag3^L/L‐YFP^ and Lag3^L/L‐N‐/−^ mice in the Y‐maze. We did not find significant behavioral deficits in the Y‐maze test by Tau PFF in either group, consistent with a prior study^[^ ^19^ ^]^ (Figure S12c–e, Supporting Information).

## 3 Conclusion

**Figure 6:**
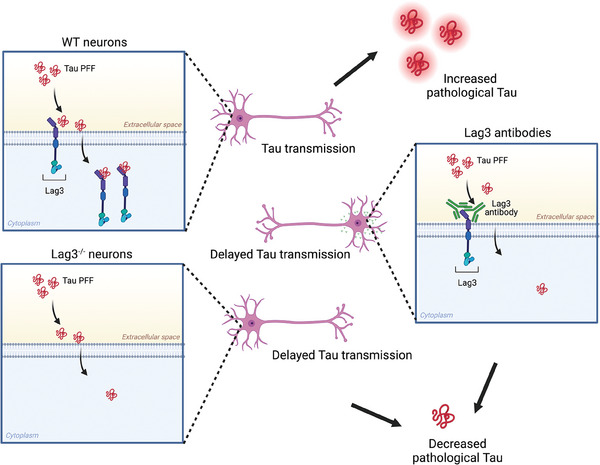
Proposed Model. Conditional Lag3 deletion or antibody‐dependent inhibition delays Tau PFF transmission: Tau PFF binding to Lag3 promotes transmission. Binding and endocytosis of Tau PFF are significantly reduced upon Lag3^−/−^ or binding to Lag3 antibodies, leading to delayed pathological transmission and toxicity (Image is prepared with Biorender).

Our prior study established that Lag3 functions as a receptor for α‐synuclein PFF. In the current investigation, we have ascertained that Lag3 also operates as a receptor for Tau PFF, which is required for neuronal uptake and transmission. Although Tau has other receptors, Lag3 is a receptor that is specific for fibrillar Tau. Using Lag3 antibodies and genetic depletion of Lag3, Tau PFF‐induced pathology and subsequent transmission were reduced. Furthermore, both Lag3^−/−^ neurons and Lag3 neuronal conditional knockout mice exhibit significantly reduced spread of Tau pathology, and Lag3 neuronal conditional knockout mice had reduced behavioral deficits (**Figure** **6**).

There has been the suggestion that Lag3 is not expressed on neurons.^[^ ^21^ ^]^ This contrasts with other studies that provide evidence for the expression of Lag3 on neurons.^[^ ^7^, ^16^ ^]^ The difference is likely due to methodological issues and the lack of optimization of thresholds to detect the low levels of Lag3 expression in neurons.

LRP1 belongs to low‐density lipoprotein receptors that work in conjunction with HSPGs.^[^ ^22^ ^]^ HSPGs and LRP1 have been identified as the receptors that bind Tau monomer, oligomer, and PFF.^[^ ^3^, ^4^ ^]^ Deletion of LRP1 can block the uptake and transmission of soluble non‐aggregated Tau in the adeno‐associated virus‐transduced WT or P301L Tau models of Tau transmission.^[^ ^2e^ ^]^ The uptake of soluble Tau seems to occur via macropinocytosis,^[^ ^23^ ^]^ but the neuronal uptake of pathogenic Tau aggregates may rely on endocytosis. In this study, we show that Tau fibrils, but not monomers of Tau, can specifically bind to Lag3 and that neuronal Lag3 is required for the endocytosis of Tau PFF. Emerging studies have shown the expression of these receptors is on neurons, microglia, and astroglia, where they exert diverse functions.^[^ ^3^, ^4^, ^6^, ^7^, ^24^ ^]^ It would be valuable to determine the roles of these Tau receptors in different cell types.

Heparin‐induced Tau fibrils were thought to resemble AD paired helical filaments (PHFs)^[^ ^25^ ^]^ with strong seeding ability in cellular and animal models overexpressing human mutant Tau; however, hep‐Tau PFF can only induce low levels of Tau pathology in non‐transgenic mice^[^ ^2c,d^ ^]^ which is likely due to different structures.^[^ ^26^ ^]^ Consistent with this notion is the observation that the structures of hep‐Tau PFF are polymorphic and differ from those in AD patients.^[^ ^26^ ^]^ Our previous studies failed to see the interaction between hep‐Tau PFF and Lag3.^[^ ^6^ ^]^ In contrast to our prior study, we also observed Aβ PFF binding SH‐SY5Y cells,^[^ ^6^ ^]^ which is likely due to the low non‐specific binding of Aβ PFF to empty vector transfected SH‐SY5Y cells in this report. These results, taken together, suggest that Lag3 is a receptor for Tau PFF, Aβ PFF, and α‐syn PFF and may indicate a more generalizable role for Lag3 in binding fibrils with amyloid‐like structure.

These results indicate that the conformation of AD‐relevant Tau seeds is critical for prion‐like propagation, and the Tau PFF‐specific receptors (i.e., Lag3) that recognize the pathogenic conformation are required. It is noted that different Tau species (oligomer, PFF, and large fibrils) and strains can cause different spreading patterns.^[^ ^2^, ^27^ ^]^ It will be interesting to further study the interaction between these receptors and Tau species/strains.

The interaction between Lag3 and Tau PFF provides a new target for therapeutic development in AD and related Tauopathies. Repurposing LAG3‐blocking antibody combination therapy has been approved by the US FDA with PD‐1 inhibition^[^ ^28^ ^]^ and could be used for neurodegenerative diseases characterized by pathologic tau and α‐syn.^[^ ^6^, ^7^ ^]^

## 4 Experimental Section

### Recombinant Tau Monomer Purification and Endotoxin Removal

Recombinant Tau was purified as previously described.^[^ ^2d^ ^]^ WT human Tau isoform (T40, 2N4R) with 441 amino acid residues was cloned into the bacterial expression vector pRK172 provided by Dr. Virginia Lee.^[^ ^2d^ ^]^ Tau was produced in *E. coli* BL21(DE3) strain (Invitrogen, Grand Island, NY, USA). Bacteria were grown in LB broth containing ampicillin at 37 °C. Isopropyl β‐D‐1‐thiogalactopyranoside (1 mm) was used to induce protein generation when the OD reached 0.8 to 1. After 2 h induction, bacteria were pelleted by centrifugation. High‐salt RAB buffer (0.1 m MES, 1 mm ethylene glycol‐bis(2‐aminoethylether)‐*N*,*N*,*N*′,*N*′‐tetraacetic acid (EGTA), 0.5 mm MgSO4, 0.75 m NaCl, 0.02 m NaF, 1 mm phenylmethylsulfonyl fluoride (PMSF), 0.1% protease inhibitor cocktail [10 ug mL^−1^ each of pepstatin A, leupeptin, tosyl phenylalanyl chloromethyl ketone, tosyl‐l‐lysyl‐chloromethane hydrochloride, and soybean trypsin inhibitor and 100 mm ethylenediaminetetraacetic acid (EDTA)], pH 7.0) was used to resuspend the pellet and followed incubation on ice for 1 h after homogenization. Then, cell lysates were boiled for 15 min, followed by rapid cooling (on ice for 30 min) and centrifuging (at 30 000 × *g* for 30 min). Supernatants were harvested and dialyzed into buffer A (20 mm piperazine‐*N*,*N*′‐bis(2‐ethanesulfonic acid), 10 mm NaCl, 1 mm EGTA, 1 mm MgSO4, 2 mm dithiothreitol (DTT), 0.1 mm PMSF, pH 6.5), then separated by fast protein liquid chromatography system using HiTrap SP HP column (GE Healthcare, Life Sciences, Wauwatosa, WI, USA). Sodium dodecyl sulfate‐polyacrylamide gel electrophoresis followed by Coomassie Blue R‐250 staining was performed to verify the presence and purity of Tau protein. Proteins were concentrated and exchanged with phosphate‐buffered saline (PBS) pH7.4 using an Amicon YM‐10 centrifuge concentrator (Millipore, Bed‐ford, MA).

Pierce high‐capacity endotoxin removal spin 0.5 mL column (Thermo Fisher Scientific, Waltham, MA, USA) was used for endotoxin removal. Briefly, 5 mL of ultrafiltrated phage lysate in SM buffer supplemented with 400 mm, NaCl, or 2 mL of AEX‐purified and ultrafiltrated phage in the same buffer were applied in the column and incubated at 4 °C for 1 h. Approximately 4.8 mL of the phage solution was collected by centrifuging at 500 × *g* at room temperature (RT) for 1 min. Next, 0.2 n NaOH in 95% ethanol was used to regenerate the column for 1–2 h at RT. After centrifuging at 500 × *g* for 1 min, the solution was discarded and followed by resuspension of resin with 2 m NaCl and endotoxin‐free ultrapure water separately. Then PBS pH 7.4 (endotoxin‐free) was used to suspend the resin for a total of three times. Purified Tau monomer protein (≈10 mg) was loaded into the column and incubated at 4 °C for 1 h, followed by collection through a centrifuge at 500 × *g* for 1 min. The quantitation of endotoxin levels in Tau protein after endotoxin removal was measured by using a Pierce chromogenic endotoxin quant kit (Thermo Fisher Scientific, Waltham, MA, USA).

### Preparation and Characterization of Tau PFF

In vitro, fibrillization of purified recombinant Tau was prepared by mixing 2 mm DTT with 300 µm recombinant Tau in 100 mm sodium acetate buffer (pH 7.0) under constant orbital agitation (1000 rpm) at 37 °C for 7 days.^[^ ^29^ ^]^ Successful fibrillization was confirmed using the thioflavin T fluorescence assay and TEM. The fibrils were mechanically broken down into small fragments by sonication (60 s, ≈0.5 s per pulse, 10% amplitude). Sulfo‐NHS‐LC‐Biotin was used to label Tau, as described previously.^[^ ^6a^ ^]^

For the extraction of Tau aggregates from AD brains, human brain tissues from two AD patients were obtained from the Division of Neuropathology, Department of Pathology at Johns Hopkins School of Medicine. Tissue was kept at −80 °C. The diagnosis of both cases was histologically confirmed. The purification of AD PHFs, AD‐Tau, was based on a previously published protocol.^[^ ^2d^ ^]^ Briefly, nine volumes v/w of high‐salt buffer (10 mm Tris‐HCl, pH 7.4, 0.8 m NaCl, 1 mm EDTA, and 2 mm DTT, with protease inhibitor cocktail, a phosphatase inhibitor, and PMSF) with 0.1% sarkosyl and 10% sucrose were added to about 2–4 g frozen frontal cortical gray matter, followed by homogenization and centrifugation at 10 000 × *g* for 10 min at 4 °C. The same buffer was used to re‐extract the pellets two times. All three initial extractions were pooled and filtered, and additional sarkosyl was added to reach 1%. The collected supernatant was centrifuged (300 000 × *g* for 60 min at 4 °C) again after 1 h nutation at room temperature. The pellets (1% sarkosyl‐insoluble) containing pathological Tau were washed once in PBS, resuspended, and passed through a 0.5 mL TB Syringe with 27 Gauge 1/2 inch permanently attached needles in PBS (≈100 µL g^−1^ gray matter). Further purification through brief sonication (20 pulses at ≈0.5 s per pulse) and centrifugation (100 000 × *g* for 30 min at 4 °C) was used for the resuspended sarkosyl‐insoluble pellets. Then, the pellets were resuspended in PBS at one half of the precentrifugation volume, sonicated with 20–60 pulses (≈0.5 s per pulse), boiled for 10 min and spun at 10 000 × *g* for 30 min at 4 °C to remove large debris. The final supernatants were collected as the final enriched AD extracts.

The in vitro fibrillization of recombinant Tau by AD‐Tau seeds was performed following a previous protocol.^[^ ^2d^ ^]^ Briefly, 4 µm AD‐Tau was used as seeds for a total concentration of 36 µm Tau in DPBS (endotoxin‐free), and the mixture was kept on an agitator for 3–4 days (1000 rpm, 37 °C). The pellets were collected after centrifugation (100 000 × *g* for 30 min at 4 °C), washed once in DPBS (endotoxin‐free) resuspended in DPBS (endotoxin‐free), and were aliquoted and stored at −80 °C referred to as Tau PFF. Right before each use, the aliquots were briefly sonicated with 30 pulses (≈0.5 s per pulse) and spun at 10 000 × *g* for 15 min at 4 °C to remove large debris.

### ThT Fluorescence Assay

To generate tau fibrils, a solution of 2 mg mL Tau in PBS, pH 7.4 incubated at 37 °C for 7 days at 1000 rpm. Then tau monomers, fibrils, and sonicated fibrils with a final concentration of 20 µm were mixed with ThT (10 µm). The reaction mixture was then added into a clear bottom black 96 well plate (Invitrogen, Cat No. M33089). The samples were excited at 450 nm, and emission was scanned between 470 and 700 nm using a Varioskan lux (Thermo scientific) multimode microplate reader. All measurements were performed in triplicate.

### TEM Measurements

Negative stain electron microscopy was used to verify the formation of fibrils for Tau PFF. Briefly, formvar carbon coated TEM grids were drop casted with 10 µL of Tau PFF for 2 min at room temperature, rinsed with water and excess water soaked with lint‐free filter paper, and then stained with 2% w/v uranyl acetate for another 1 min. The stain was blotted off with lint‐free filter paper. Grids were allowed to dry completely before taking images captured on Hitachi TEM operating at 80 kV.

### Primary Neuronal Cultures, Tau PFF Administration, and Neuron Binding Assays

Primary cortical neurons were prepared from E15.5 pups of C57BL/6 mice and cultured in neurobasal media adding with B‐27, 0.5 mm l‐glutamine, streptomycin, and penicillin (Corning, Glendale, AZ, USA) on tissue culture plate coated with poly‐l‐lysine. The medium was changed every 3 days. At 7 DIV, Tau PFF (2 µg mL^−1^) was added and incubated for 14 days in the medium. Subsequently, biochemical experiments and pathology assays were performed. Tau‐biotin PFF at different concentrations were utilized to define the amount of bound Tau‐biotin PFF to WT and Lag3^−/−^ neurons. ImageJ software was used for the quantitative analysis.

### SH‐SY5Y Cell Surface Binding Assays

SH‐SY5Y cells were transfected with Lag3 expressing plasmid with Lipofectamine 2000 per the manufacturer's instruction. Transfected cells were treated with different concentrations of monomer equivalent Tau‐biotin PFF in DMEM media (10% FBS) for 2 h at RT. Unbound Tau‐biotin PFF was removed by thoroughly washing (five times, 20 min each) with DMEM media (10% FBS) on a horizontal shaker, followed by fixation with 4% paraformaldehyde in PBS. The cells were washed three times with PBS, followed by blocking with PBS containing 10% horse serum and 0.1% Triton X‐100 for 30 min. After blocking, the cells were incubated with alkaline‐phosphatase–conjugated streptavidin (1:2000 dilution) in PBS with 5% horse serum and 0.05% Triton X‐100 for 16 h. Alkaline phosphatase signal was detected histochemically by 5‐bromo‐4‐chloro‐3‐indolyl phosphate/nitro blue tetrazolium reaction.^[^ ^8^ ^]^ Images were acquired on Zeiss Axiovert 200 m microscope. The intensity of bound Tau‐biotin PFF to Lag3‐transfected SH‐SY5Y cells was quantified with ImageJ software. Desired range of intensity values and background exclusion for control was acquired by adjusting the threshold under the Image/Adjust menu. The same threshold settings were applied to all images in each experiment. The binding curve and KD values were calculated with GraphPad Prism software.

### Plasmids and Deletion Mutants

Human LAG3 and mouse Lag3 cDNA clones were kindly obtained from Dr. Charles Drake at the Johns Hopkins University School of Medicine. Lag3 deletion mutants (D1, D2, D3, D4, TM, and ICD) with a HA tag and deletion mutants of Lag3 D1 domain with a myc tag were constructed by PCR with In‐Fusion HD Cloning Plus systems (Takara; Bio Inc., Otsu, Japan) using the CloneAmp HiFi PCR Premix (a high‐fidelity PCR polymerase included with all In‐Fusion HD Cloning Plus Systems), and primers with a 15 bp overlap at their 5′ ends to incorporate the mutation of interest. The DNA was separated on a 1% agarose gel, and the appropriate band was excised and isolated using a gel extraction kit (Qiagen, Valencia, CA, USA). In‐Fusion HD enzyme premix was added to the linearized PCR product and transformed into the Stellar Competent Cells (Takara; Bio Inc., Otsu, Japan). The integrity of plasmid sequences was verified through sequencing.

### Lentiviral Vector Construction, Production, and Transduction

The preparation of lentiviral vectors was performed based on a prior publication.^[^ ^30^ ^]^ Briefly, Lag3 or Lag3 deleted mutations with HA tag were subcloned into a lentiviral cFugw vector through the Age I restriction enzyme site. The human ubiquitin C promoter was utilized to drive gene expression. The recombinant cFugw vector gathering with three packaging vectors (pLP1, pLP2, and pVSV‐G (1.3:1.5:1:1.5)) was transiently transfected into HEK293FT cells and the lentiviruses were produced. The viral supernatants were concentrated and collected through ultracentrifugation (50 000 × *g*, 3 h) after 48 and 72 h transfection. Viral particles were stored in a serum‐free medium at −80 °C. Neurons (DIV 4 to 5) were transducted by lentivirus separately carrying Lag3, Lag3 deletion mutants for 72 h, while empty vectors served as a control (1 × 109 transduction units (TU) mL^−1^).

### Live Images

Primary cultured neurons were cultured on 12 mm coverslip in a chamber (RC‐48LP, Warner Instruments) with physiological saline solution (40 mm NaCl, 5.4 mm KCl, 1.3 CaCl_2_ mm, 1.0 mm MgCl_2_, 20 mm glucose, and 25 mm hydroxyethyl piperazine ethane sulfonic acid, pH to 7.4) and imaged using Microscope Axio Observer Z1 (Zeiss, Dublin, CA, USA). Tau PFF was labeled with pHrodo red (Invitrogen, Grand Island, NY, USA). pHrodo red fluorescence increases as the pH drops when it gets into neurons. Tau PFF‐pHrodo was directly added to Lag3 WT and Lag3^−/−^ neuron groups. For the WT + Lag3 and Lag3^−/−^ + Lag3 groups, neurons were transduced with lentivirus carrying Lag3 (empty vector as a control). Live images were recorded every 0.5–1 min for 90–100 min. 1–4 min later after 0.5 µm Tau PFF‐pHrodo treatment, the cells were appropriate for recording. Fluorescence intensity of the neuron at 2–3 min after Tau PFF‐pHrodo treatment was used as the baseline background. Quantification was performed using the Zeiss Zen Software to outline the neurons and subtract the background. The percentage of internalized Tau PFF‐pHrodo signal at each time point was calculated based on the ratio to the baseline in each experiment.

### Calcium Imaging

Calcium imaging was utilized to visualize calcium signaling in a living neuron directly through intracellular calcium flux. In this study, Fluo‐2 acetoxymethyl (AM) ester (Thermo Fisher Scientific, Waltham, MA, USA) was used as the fluorescent calcium indicator to monitor the intracellular calcium levels in primary cultured cortical neurons. Primary cortical neurons obtained from WT and Lag3^−/−^ embryo mouse cortex were plated on coverslips coated with polyornithine and incubated for 14 days. Right before imaging, the cells were treated with Fluo‐2 AM for 30 min at 1 µm final concentration. Fura‐2–free PSS buffer was used to wash the cells; then the neurons were put in a chamber (RC‐48LP, Warner Instruments) with a 37 °C heated adaptor on the Microscope Axio Observer Z1 (Zeiss, Dublin, CA, USA). The field containing more than ten neurons was selected as regions of interest. The imaging was conducted with an excitation/emission wavelength of 485/525 nm. After the baseline fluorescent signals were kept steady for 5 min, 500 nm Tau PFF was added and the imaging recording lasted for another 90 min. During the imaging recording process, images were captured every 30 s and analyzed by Image J and Zen software (Carl Zeiss, Germany).

### Mouse Strains

C57BL/6 wild‐type (WT) (strain 000664) mice were purchased from the Jackson Laboratories (Bar Harbor, ME), while Lag3^−/−^ mice were kindly provided by Dr. Charles Drake (Johns Hopkins University School of Medicine) maintained on a C57BL6 background. Lag3^L/L‐YFP^ mice have been previously described.^[^ ^17^ ^]^ Nestin^Cre^ mice^[^ ^18^ ^]^ were obtained from the Jax lab (strain 003771). Lag3 neuronal conditional knockout (Lag3^L/L‐N‐/−^) mice was obtained by breeding Lag3^L/L‐YFP^ conditional knockout‐reporter mice^[^ ^17^ ^]^ Lag3^L/L‐YFP^ with nestin^Cre^ mice (Jax Lab, strain: 0 03771).^[^ ^18^ ^]^ These mice did not develop any autoimmune or inflammatory phenotype. The research was approved by the Johns Hopkins University Animal Care and Use Committee and all procedures were completed according to the NIH Guide for the Care and Use of Experimental Animals (NIH Publication No. 85‐23, revised 1996). Animals were housed with free access to water and food at a 12:12 h dark–light cycle. Before performing any animal experiments, all mice were kept in the procedure room for an acclimatization period.

### Lag3 Antibody Blocking Experiments

Anti‐Lag3 antibodies (410C9, C9B7W, 17B4) were added to the cell cultures (30 nm) 30 min before the Tau PFF treatment, while the rat IgG (Invitrogen, Carlsbad, CA, USA) and mouse IgG (Santa Cruz, Dallas, TX, USA) were used as negative controls. SH‐SY5Y cells that overexpressed Lag3 for the binding assay were used 2 days after Lag3 transfection. For the endocytosis endosome enrichment assay, mouse primary cortical neurons were cultured for 12 days (12 DIV) before treatment. For Tau pathology and transmission experiments, Tau PFF was added to wells of primary cultured neurons cultured for 7 days in vitro (7 DIV). In the neuron transmission experiment, the antibodies were added to the middle chamber (chamber 2) of the microfluidic chamber system.

### Isolation of Tau Oligomers with Sucrose Gradient Fractionation

Tau oligomers were prepared as described previously by Comb et al.^[^ ^9^ ^]^ Briefly, a sucrose gradient was prepared in a polycarbonate tube by layering sucrose in descending order of concentration, starting from the most concentrated 50% at the bottom, followed by 40%, 30%, and 20% at the top. The final layer consisted of tau aggregates placed undisturbed on top of the sucrose gradient. The sample was then centrifuged in a TLA120 rotor at 51 000 rpm (172894 RCF [avg]; 222854 RCF [max]) for 2 h at 4 °C. Following centrifugation, each layer was separated carefully in a new tube. Further purification of oligomeric tau was performed with the collected fraction by pelleting and resuspending in a PBS buffer. The resuspended pellet was further centrifuged at 55 000 rpm for 3 h at 4 °C using a TLA120 rotor. Post‐centrifugation, the supernatant was discarded, and the pellet was resuspended in a 100 µL PBS. This pellet, rich in the presence of oligomeric tau, was further verified using TEM. The top sample layer displayed minimal to no Tau, while the fraction with 20% sucrose concentration contained relatively low amounts of Tau. As expected, the 30% sucrose layer exhibited a pronounced concentration of oligomeric tau structures. The 40% sucrose fraction presented a combination of oligomeric tau and short tau filaments, while the fraction with 50% sucrose predominantly contained a range from short to long tau filaments.

### Purification of Tau Seeds from PSP and PiD Brain Tissues

Pathological Tau seeds from human brain tissue were isolated as described by Guo et al.^[^ ^2d^ ^]^ Briefly, Human brain tissues from PSP and PiD patients with tau pathology were provided by Dr. Juan C. Troncoso at Johns Hopkins University Brain Resource Center. The use of postmortem brain tissues for research was approved by John Hopkin's Institutional Review Board with informed consent from patients or their families. For each purification, 100 mg of frontal cortical gray matter was homogenized in 900 µL of high‐salt buffer (10 mm Tris‐HCl, pH 7.4, 0.8 m NaCl, 1 mm EDTA, and 2 mm DTT, with protease and phosphatase inhibitor cocktail) with 0.1% sarkosyl and 10% sucrose. Homogenized tissues were centrifuged at 10 000 × *g* for 10 min at 4 °C. Pellets were re‐extracted twice in the same buffer conditions. The supernatants from three extractions were pooled together and filtered. Additional sarkosyl was added to the pooled sample to obtain 1% final sarkosyl concentration. Samples were nutated for 1 h at room temperature followed by centrifugation at 300 000 × *g* for 1 h at 4 °C. The pellet containing 1% sarkosyl‐insoluble pathological Tau was collected and washed and resuspended in 100 µL PBS by passing through 27G 0.5 in. needles. The resuspended pellets were further sonicated (20 pulses at ≈0.5 s per pulse) and centrifugation at 100 000 × *g* for 30 min at 4 °C. After centrifugation, pellets containing 60–70% of tau were resuspended in PBS at 60 µL PBS, sonicated with 20–60 short pulses (≈0.5 s per pulse), and centrifuged at 10 000 × *g* for 30 min at 4 °C to remove large debris. The final supernatant containing enriched pathological Tau seeds was collected and used for further experiments to prepare PSP and PiD seeded Tau PFF.

### Microfluidic Chambers

Triple compartment microfluidic devices (TCND1000, Xona Microfluidic, LLC, Temecula, CA, USA) were used for the Tau transmission experiment in vitro. After the glass coverslips were prepared and coated as previously described, they were attached to the microfluidic device.^[^ ^31^ ^]^ About 100,000 primary cultured neurons were plated in each chamber of the device. Lentivirus Lag3 was used to transduce WT and Lag3^−/−^ neurons (4 DIV) to create WT + Lag3 and Lag3^−/−^ + Lag3 neurons. Then, Tau PFF (1 µg mL) was added into chamber 1 at 7 DIV. In order to control the direction of medium flow, the difference of medium volume (between chamber 1 and chamber 2, and between chamber 2 and chamber 3), was maintained at 75 µL based on the manufacturer's instructions. After 14 days of treatment, neurons were fixed using 4% paraformaldehyde in PBS, followed by immunofluorescence staining.

### Stereotaxic Injection Procedure

Aliquoted Tau PFF was used in this experiment. Right before stereotaxic injection, the Tau PFF was diluted with PBS and briefly sonicated in a temperate controlled water bath sonicator (≈0.5 s per pulse, 30 pulses) and spun at 10 000 × *g* for 15 min at 4 °C to remove large debris. Pentobarbital was used for the anesthesia of 3 months Lag3^L/L‐YFP^ and Lag3 neuronal conditional knockout (Lag3^L/L‐N‐/−^) mice. Two µL Tau PFF (1 µg µL^−1^) were delivered into two sides of the dorsal hippocampus and overlaying cortex using the stereotactic apparatus at reference coordinates for the hippocampus (Bregma −2.54 mm, 2 mm from midline, and −2.4 mm from skull) and the cortex (Bregma −2.54 mm, 2 mm from midline, and −1.4 mm from skull) based on previous publication.^[^ ^2d^ ^]^ Hamilton syringe (2 µL, Hamilton, Reno, NV, USA) was used for the injections with a rate of 0.1 µL min^−1^, and the needle was kept in place for ≥5 min before gentle removal. Animals were carefully monitored and taken care of after the operation. Animal behavior tests were performed at 9 months after injection. After euthanization, the mice were used for neurochemical, biochemical, and histological studies. Tissues were immediately frozen after removal and stored at −80 °C for biochemical experiments. For immunofluorescent staining studies, mice were perfused transcardially with PBS followed by fixation with 4% paraformaldehyde (PFA), and brains were removed and fixed overnight in 4% PFA, followed by keeping in 30% sucrose in PBS until tissue settled down.

### Immunofluorescence and Mapping of Tau Pathology

Serial brain sections (40 µm thick) were used to perform immunohistochemistry and immunohistofluorescence. Coronal sections were incubated in primary antibodies for P‐Tau (AT8) (Invitrogen, Grand Island, NY, USA). Immunoreactivity was labeled using appropriate fluorescent secondary antibodies conjugated to Alexa‐fluor 568 (Invitrogen, Carlsbad, CA, USA). DAPI was used together with secondary antibodies to stain the nuclei. Images (IF) were detected by confocal scanning microscopy (LSM 880, Zeiss, Dublin, CA, USA) or fluorescent microscope (BZ‐X710, Keyence, Osaka, Japan).

### Behavioral Analysis

Behavior tests, such as open field, Y‐maze, social interaction tests were used to assess behavioral deficits in mice at 9 months after the injection of PBS control or Tau PFF. The experiment was performed with a blinded treatment group for all behavioral tests. All tests were performed between 13:00 and17:00 in the lights‐on cycle.

### Open Field Test

General motor activity and anxiety‐related behavior were assessed by an open field test. In brief, all mice were randomized by four open field chambers to mix up control versus experimental groups. Then, the mice were directly placed into the middle of the open field (a rectangular plastic box, 40 cm × 40 cm × 40 cm, length × width × height). The plastic box was divided into 36 identical sectors, and the field was subdivided into peripheral and central sectors.^[^ ^32^ ^]^ Movements were recorded for 5 min using a digital camera under dim light. The open field was wiped down with Vimoba after the removal of each experimental mouse. Distance traveled was recorded using the Photobeam Activity System for the analysis of locomotor activity, and the percentage of time spent in the center square was measured for anxiety analysis.

### Y‐Maze

We used Y‐maze to evaluate the spatial working memory of mice. The experiment consisted of the training and test phases were performed as previously described.^[^ ^33^ ^]^ Briefly, the mice were collected from their rooms and brought to the behavioral suite to allow for acclimation. The Y‐maze apparatus consists of three symmetrical arms with an angle of 120° between adjacent arms (15 cm × 10 cm × 40 cm, height × width × length, each arm A, B, and C). The three arms were randomly allocated for the start arm, familiar arm, and novel arm. During the training phase, mice were allowed to explore start arm and familiar arm freely for 8 min while the novel arm was blocked by a removable barrier. After the training phase, the animal was removed from the Y‐maze apparatus and kept in their home cage for 2 h. Then the test was conducted while the barrier wall of the novel arm was removed. The mouse had access to all three arms freely for 8 min, starting at the same end of the start arm. Behavioral measurements were performed and analyzed by Any‐Maze software. Additionally, a spontaneous alternation was also recorded when the mouse entered the three arms in turn, such as CBA, ABC, or BAC. The time spent in each arm, the number of arms entered, and the number of alternations or triads were recorded. In addition, the relative time spent in the novel arm and the number of alternations were calculated. Finally, the total distance the mice moved on the entire maze was recorded and analyzed in order to assess general locomotor activity.

### Social Interaction Tests

Three chamber social interaction tests were conducted to evaluate the mice's sociability and social novelty. In the first phase (habituation), the mice were placed into the three‐chambered apparatus without any stranger mice, opened all the gates, and allowed the mouse to explore for 10 min. In the second phase (sociability), the gates of the chambers were closed, and the test mouse was kept in the center of the apparatus. Subsequently, the ′stranger 1″ mouse was placed under one of the wire baskets while the other basket with no animal inside was placed in the other chamber. Then, the gates were opened and it was allowed to explore for 10 min. In the third phase (preference for social novelty), both gates were closed, the ′stranger 2″ mouse was placed under the empty wire basket in the second phase, and the test mouse was allowed to explore for 10 min. The sniffing time at Stranger 1 and Stranger 2 was recorded using Topscan 3.0 software.

### Quantification and Statistical Analysis

All data were analyzed using GraphPad Prism 9. Statistics Data were presented as the mean ± SEM with at least three independent experiments. Representative morphological images were obtained from at least three experiments with similar results. Statistical significance was assessed via a one or two‐way ANOVA test followed by indicated post hoc multiple comparison analysis. Assessments with *p* < 0.05 were considered significant.

## Conflict of Interest

D.A.A.V and C.J.W. have submitted patents on Lag3 approved or pending and are entitled to a share in net income from licensing patent rights for commercial development. D.A.A.V: cofounder and stock holder—Novasenta, Potenza, Tizona, Trishula; stock holder—Oncorus, Werewolf; patents licensed and royalties—BMS, Novasenta; scientific advisory board member—Tizona, Werewolf, F‐Star, Bicara, Apeximmune, T7/Imreg Bio; consultant—BMS, Incyte, Regeneron, Ono Pharma, Avidity Partners; funding—BMS, Novasenta.

## Author Contributions

C.C. and R.K. contributed equally to this work. C.C. and R.K. led the project and contributed to all aspects of the study. H.W., X.L.Y., K.G., C.R., Y.K., A.B., S.K., D.J., L.W., A.W., R.C., and S.Z. contributed to biochemical, cellular, mouse experiments work. C.J.W. and D.A.A.V. provided the essential mice strains and reagents. O.P., D.W.N., and J.C.T. provided key brain samples. J.L.T., P.C.W., and M.Y.Y. helped with the project/experimental design. X.M., V.L.D., and T.M.D. designed research. X.M., V.L.D., and T.M.D. wrote and revised the manuscript. All authors reviewed, edited, and approved the manuscript.

## Supporting information

Supporting Information

## Data Availability

The data that support the findings of this study are available from the corresponding author upon reasonable request.
