# Supplementary material for: Lymphocyte‐Activation Gene 3 Facilitates Pathological Tau Neuron‐to‐Neuron Transmission
Source: Adv Sci (Weinh). 2024 Feb 7;11(16):2303775. doi: 10.1002/advs.202303775 (PMC11040377; doi:10.1002/advs.202303775)
Supplement: Supplementary file 1 — Supporting Information [file ADVS-11-2303775-s001.pdf]

ADVANCED SCIENCE
Open Access

Supporting Information

for *Adv. Sci.*, DOI 10.1002/advs.202303775

Lymphocyte-Activation Gene 3 Facilitates Pathological Tau Neuron-to-Neuron Transmission

Chan Chen, Ramhari Kumbhar, Hu Wang, Xiuli Yang, Kundlik Gadhave, Cyrus Rastegar, Yasuyoshi Kimura, Adam Behensky, Sumasri Kotha, Grace Kuo, Sruthi Katakam, Deok Jeong, Liang Wang, Anthony Wang, Rong Chen, Shu Zhang, Lingtao Jin, Creg J. Workman, Dario A. A. Vignali, Olga Pletinkova, Hongpeng Jia, Weiyi Peng, David W. Nauen, Philip C. Wong, Javier Redding-Ochoa, Juan C. Troncoso, Mingyao Ying, Valina L. Dawson, Ted M. Dawson* and Xiaobo Mao**

Supplementary figures and Figure legends:

Lymphocyte-activation gene 3 facilitates pathological Tau neuron-to-neuron transmission

Chan Chen[1,2,†], Ramhari Kumbhar[1,2,†], Hu Wang[1,2], Xiuli Yang[1,2], Kundlik Gadhave[1,2], Cyrus Rastegar[1,2], Yasuyoshi Kimura[1,2], Adam Behensky[1,2], Sumasri Kotha[1,2], Grace Kuo[1,2], Sruthi Katakam[1,2], Deok Jeong[1,2], Liang Wang[1,2], Anthony Wang[1,2], Rong Chen[1,2], Shu Zhang[1,2], Lingtao Jin[3], Creg J. Workman[4], Dario A.A. Vignali[4,5,6], Olga Pletinkova[7,‡], Hongpeng Jia[8], Weiyi Peng[9], David W. Nauen[7], Philip C. Wong[7], Javier Redding-Ochoa[2,7], Juan C. Troncoso[2,7], Mingyao Ying[2,10], Valina L. Dawson[1,2,11,,12,13]*, Ted M. Dawson[1,2,12,13,14]*, Xiaobo Mao[1,2,15,16]*

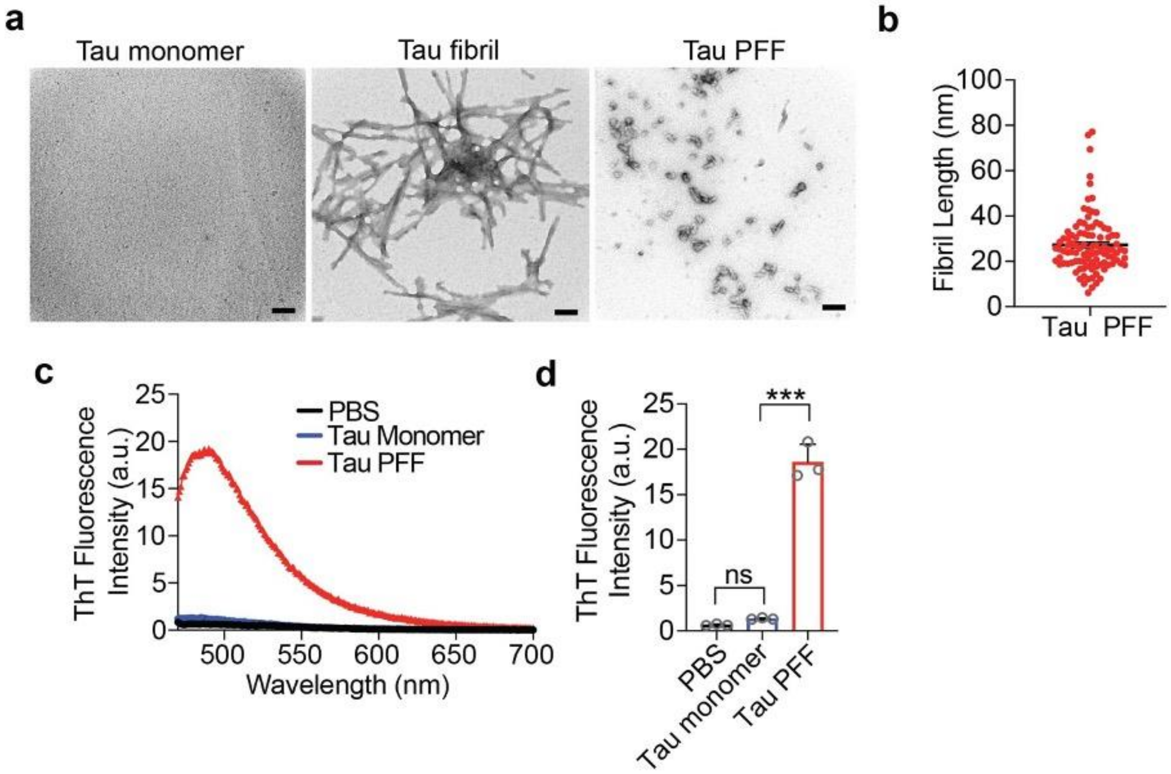

Figure S1: Characterization of Tau PFF. │ **a**. Transmission electron microscopy of Tau monomer, Tau fibrils (unsonicated) and Tau PFF (sonicated). Scale bar, 100 nm. **b.** Fibril length of sonicated Tau PFF. The error bar represents SEM. **c.** The ThT fluorescence spectra were recorded at 450 nm excitation and 470 to 700 nm emission wavelengths. **d.** ThT fluorescence intensity at 485 nm for PBS, Tau monomer, and Tau PFF (sonicated), (n = 3). Error bars represent SEM.

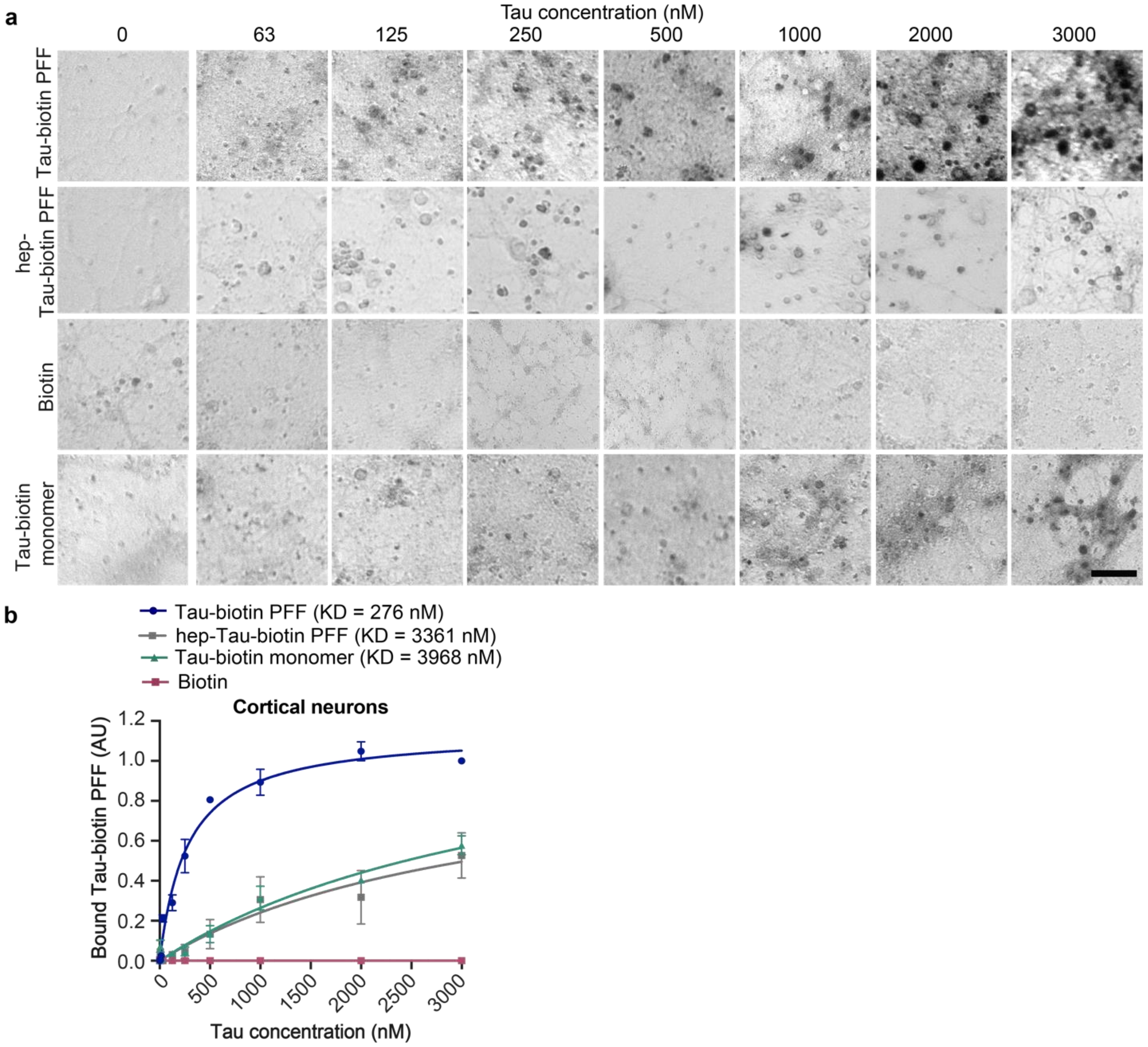

Figure S2: Binding of biotin-labeled Tau PFF to primary cortical neurons. | **a.** Representative images of Tau PFF-biotin binding to wild-type (WT) mouse primary cortical neurons, Scale bar, 100μm. **b.** Quantification of binding of biotin, Tau-biotin monomer and heparin induced Tau-biotin (hep-Tau) PFF and Tau-biotin PFF to mouse primary cortical neurons (Tau-Biotin PFF KD = 276 nM), Data are the means ± SEM, *n* = 3 independent experiments.

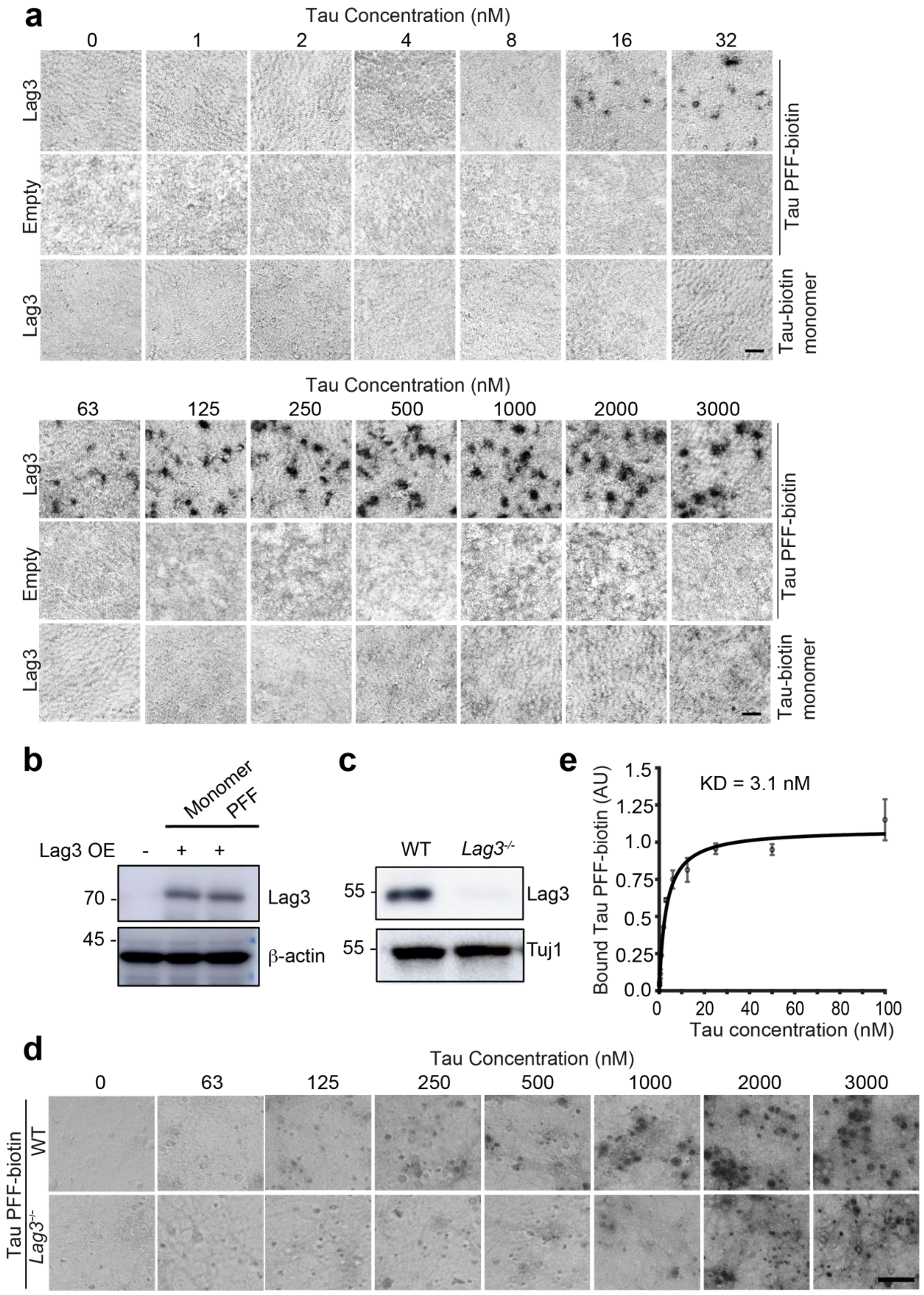

Figure S3: Biotin-labeled Tau PFF can bind to Lag3 expressing SH-SY5Y cell surface receptor. | **a.** Tau-biotin PFF binds to Lag3 overexpressing SH-SY5Y cell surface in a saturable manner, as a function of Tau biotin total concentration (monomer equivalent for PFF preparations). Scale bar, 100 µm. **b.** Immunoblot showing overexpression (OE) of Lag3 into SH-SY5Y cells in the cell surface binding assays. **c.** Immunoblot showing Lag3 expression level in WT and *Lag3* knockout (*Lag3*$^{-/-}$) mouse primary cortical neurons. **d.** Representative images of Tau PFF-biotin binding to WT and *Lag3*$^{-/-}$ mouse primary cortical neurons. Scale bar, 100 µm. **e.** Tau PFF binds to human recombinant LAG3 as assessed by ELISA. KD = 3.1 nM, *n* = 3.

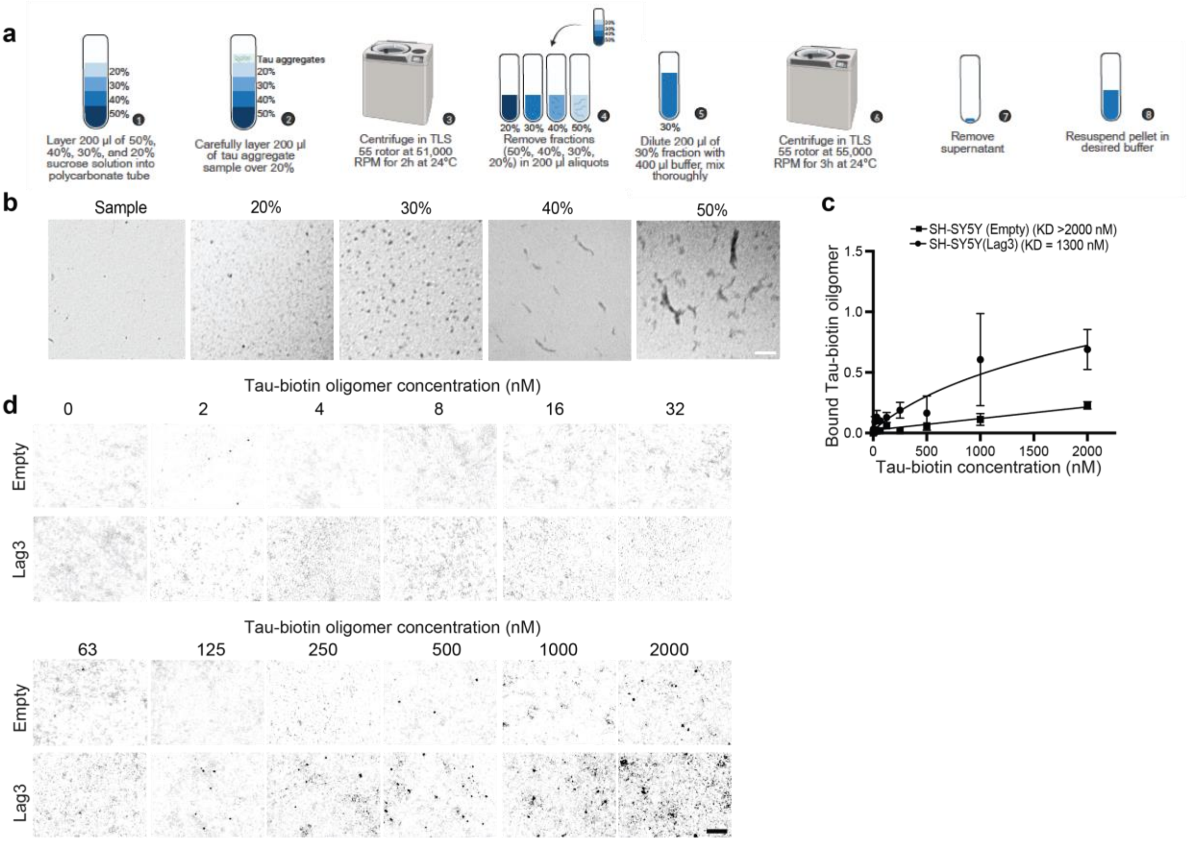

Figure S4: Tau-biotin oligomer binding to Lag3 in SH-SY5Y cells. │ **a.** Flowchart for isolation of oligomeric Tau. b. Representative TEM images of Tau oligomers obtained by sucrose gradient. Globular Tau-biotin oligomers were obtained in 30% glucose gradient. Scale bar, 100 nm. **c.** Quantification of binding of Tau-biotin oligomer to empty and Lag3 plasmid transfected SH-SY5Y cells. Data are the means ± SEM, *n* = 3 independent experiments. **d.** Representative images of Tau-biotin oligomer binding to empty and Lag3 expressing plasmid transfected SH-SY5Y cell surface. Scale bar, 100 µm.

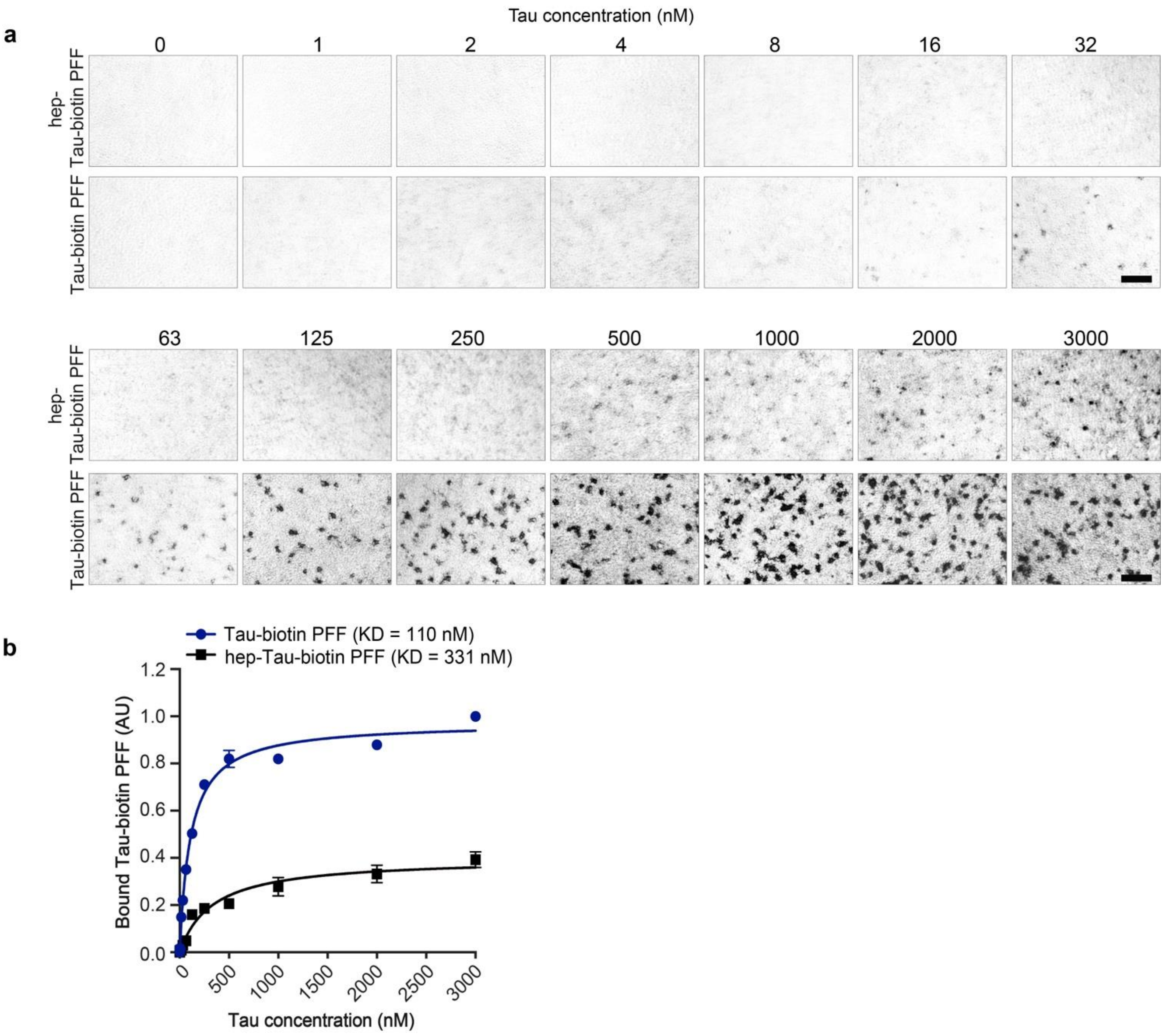

Figure S5: Heparin inhibits Tau PFF binding to Lag3. │ **a.** Representative images of Tau-biotin PFF and Heparin induced Tau (hep-Tau)-biotin PFF binding to Lag3-expressing SH-SY5Y cell surface. Scale bar, 100 µm. **b.** Quantification of binding of Tau-biotin PFF and hep-Tau PFF-biotin to SH-SY5Y cell surface. Data are the means ± SEM, *n* = 3 independent experiments.

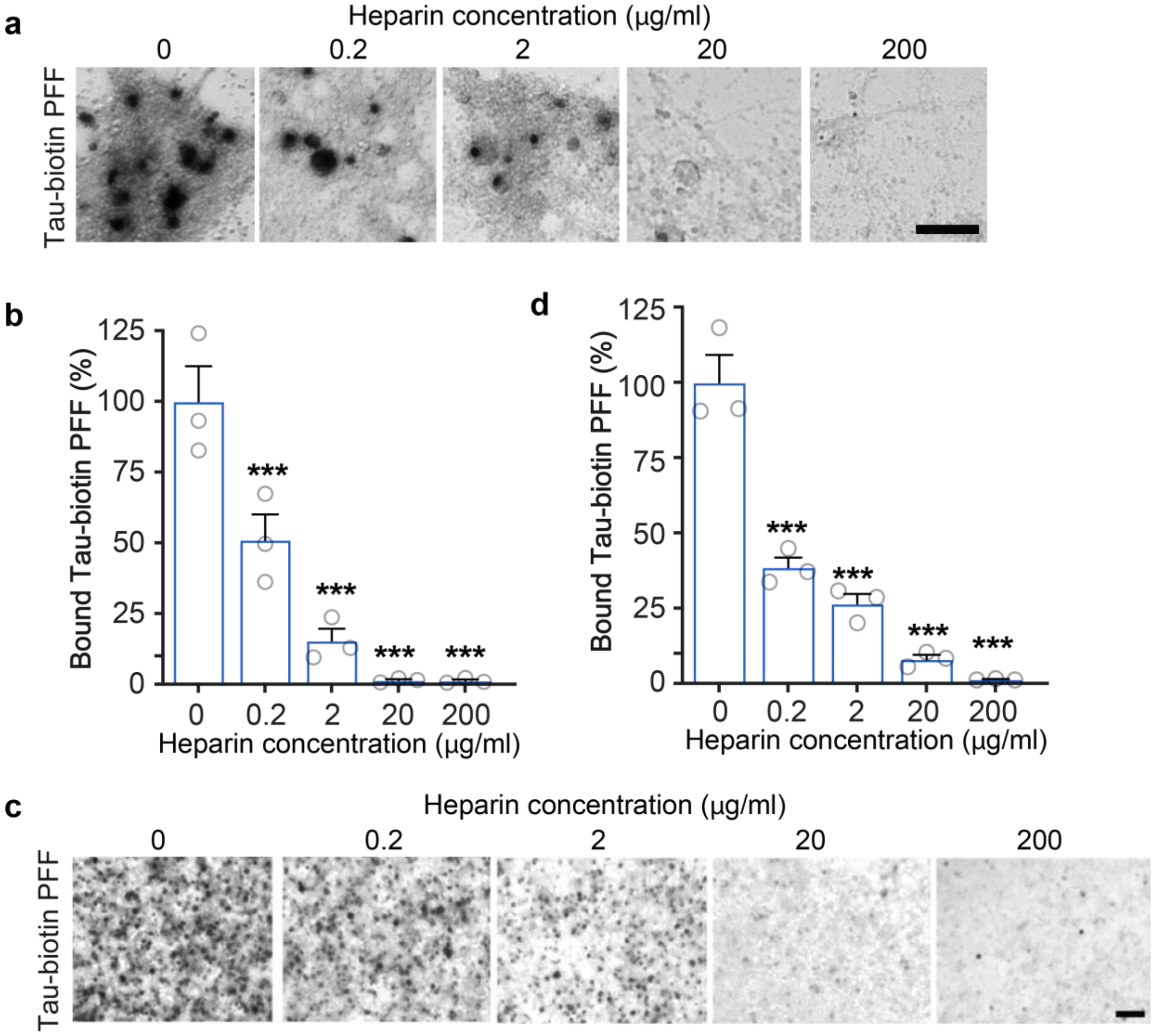

Figure S6: Heparin inhibits Tau PFF binding to Lag3 in primary cortical neurons. │ **a.** Representative images of Tau-biotin PFF binding in presence of increasing concentration of heparin (0-200 μg/ml) to cell surface of primary cortical neurons. Scale bar, 100 µm. **b.** Quantification of binding of Tau-biotin PFF in presence of heparin, data are the means ± SEM, *n* = 3 independent experiments. **c.** representative images of Tau-biotin PFF binding in presence of increasing concentration of heparin (0-200 μg/ml) to Lag3-expressing SH-SY5Y cell surface. Scale bar, 100 µm. **d.** Quantification of binding of Tau-biotin PFF in presence of heparin to SH-SY5Y cell surface. Data are the means ± SEM, *n* = 3 independent experiments.

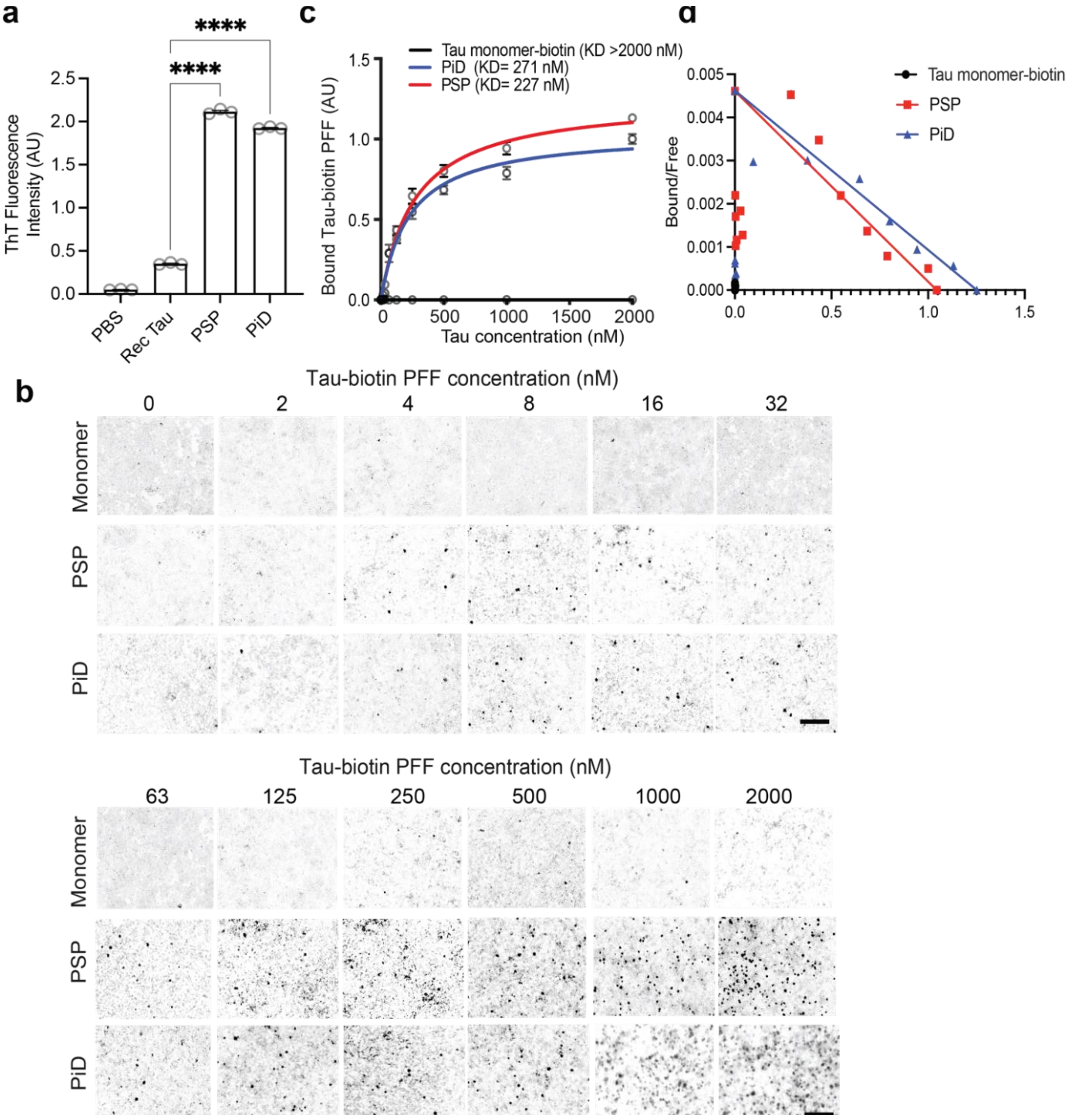

Figure S7: Tau PFF amplified from tauopathy binding to Lag3. │ **a.** ThT fluorescence intensity at 485 nm for PBS, recombinant Tau PFF (Rec Tau) and 10% PSD and PiD patient seeded recombinant Tau PFF (*n* = 3). Error bars represent SEM. **b.** Representative images of Tau-biotin PFF to Lag3-expressing SH-SY5Y cell surface at 500 nM concentration. Data are the means ± SEM, *n* = 3 independent experiments, Scale bar, 100 μm. **c.** Quantification of binding of different Tau-biotin PFF strains to Lag3 expressing SHSY-5Y cells. **d.** Scatchard analysis. Data are the means ± SEM, *n* = 3 independent experiments.

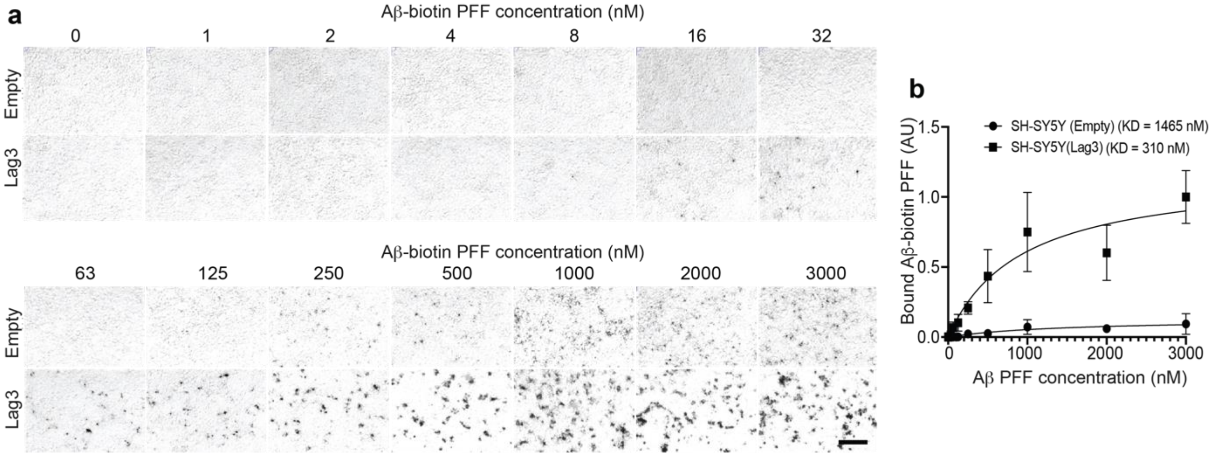

Figure S8: Aβ-biotin PFF binding to Lag3 in SH-SY5Y cells. │ **a.** Representative images of Aβ-biotin PFF binding to empty and Lag3-expressing plasmid transfected SH-SY5Y cell surface **b.** Quantification of binding of Aβ fibrils to SH-SY5Y cell surface. Data are the means ± SEM, *n* = 3 independent experiments, Scale bar, 100 μm.

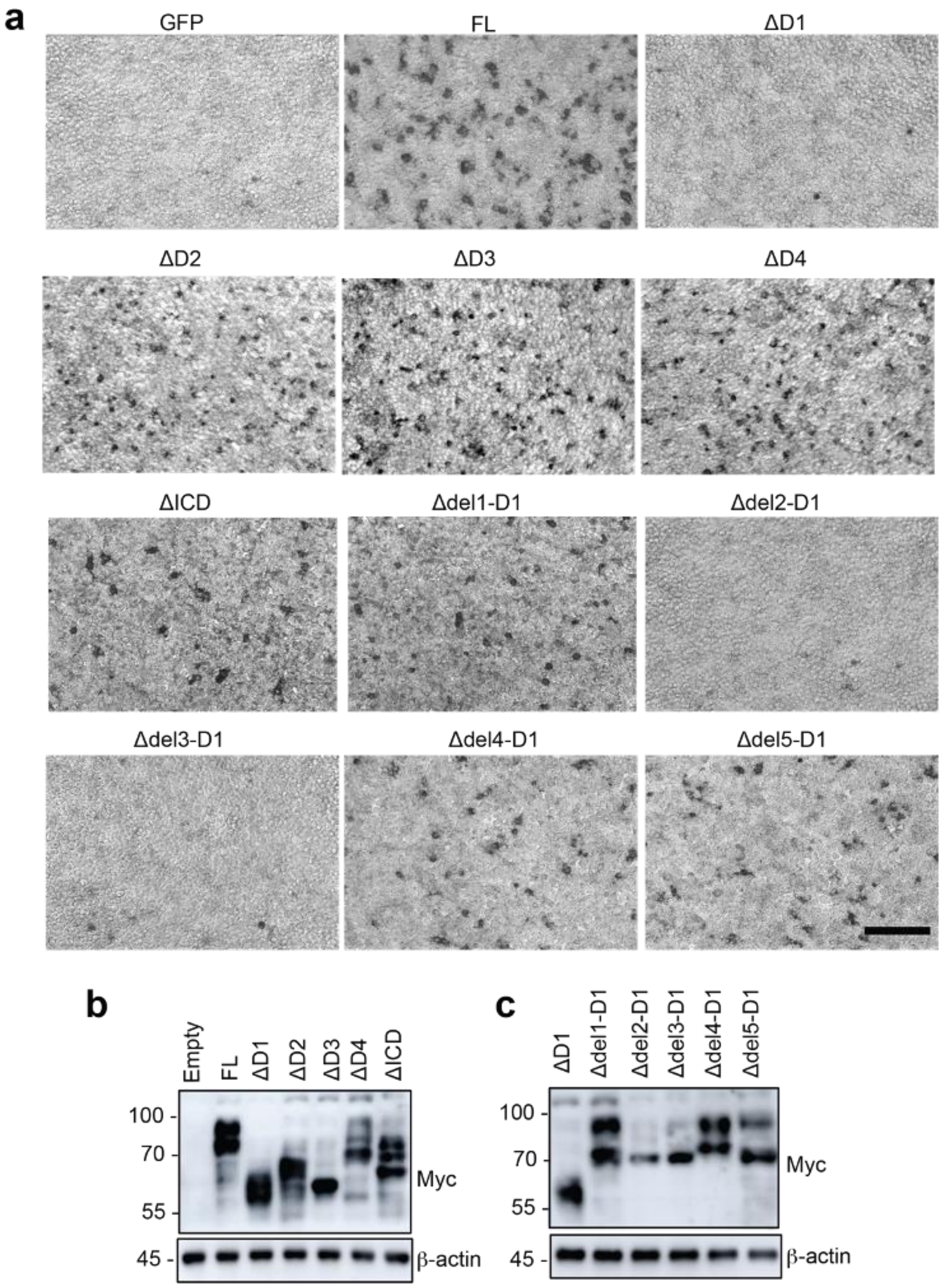

Figure S9: Lag3 deletion mutants were overexpressed into HEK293FT cells and assessed for Tau PFF-biotin binding. │ **a.** Representative images of Tau PFF-biotin binding to full-length (FL) and deletion mutants of Lag3: extracellular domains (ΔD1-ΔD4), intracellular domain (ΔICD, and subdomains of D1 domain (Δdel1-5-D1). Scale bar, 100 μm. **b-c.** Immunoblot showing overexpression levels of full-length Lag3 and deletion mutants of Lag3: extracellular domains (ΔD1-ΔD4), intracellular domain (ΔICD, and subdomains of D1 domain in HEK293FT cells used for Tau PFF-biotin binding.

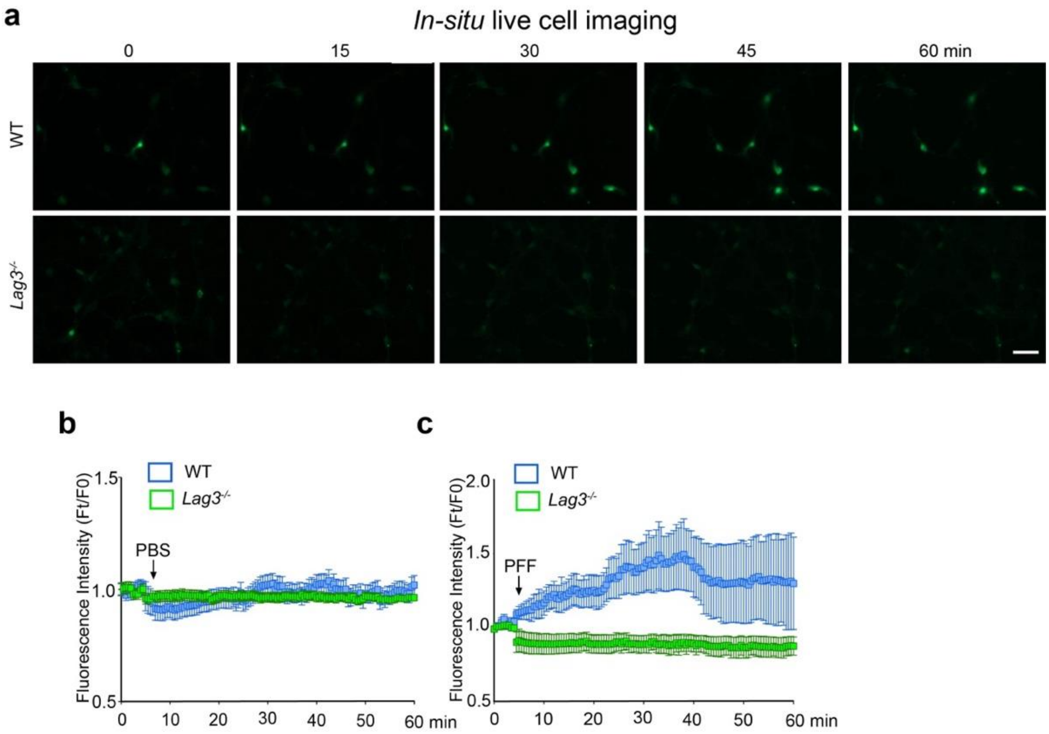

Figure S10: Tau PFF induced increase in intracellular Ca^{2+} level is mediated by Lag3. | **a.** Time series imaging of calcium-dependent fluorescence of WT and *Lag3*$^{-/-}$ neurons. Neurons were treated with 1 μM Fluo-2 acetoxymethyl (AM) ester for 30 min followed by PBS or 500 nM Tau PFF. Live images were acquired for the indicated time period. Scale bar, 50 μM. **b.** Ca^{2+} levels from WT and *Lag3*$^{-/-}$ neurons without Tau PFF treatment. **c.** Fluo-2 acetoxymethyl (AM) associated fluorometric measurement showing changes in intracellular Ca^{2+} signal in WT and *Lag3*$^{-/-}$ neurons upon Tau PFF treatment. Ft fluorescence intensity of the indicator at t minutes, F0 is Fluorescence intensity at 0 min. Error bars represent SEM.

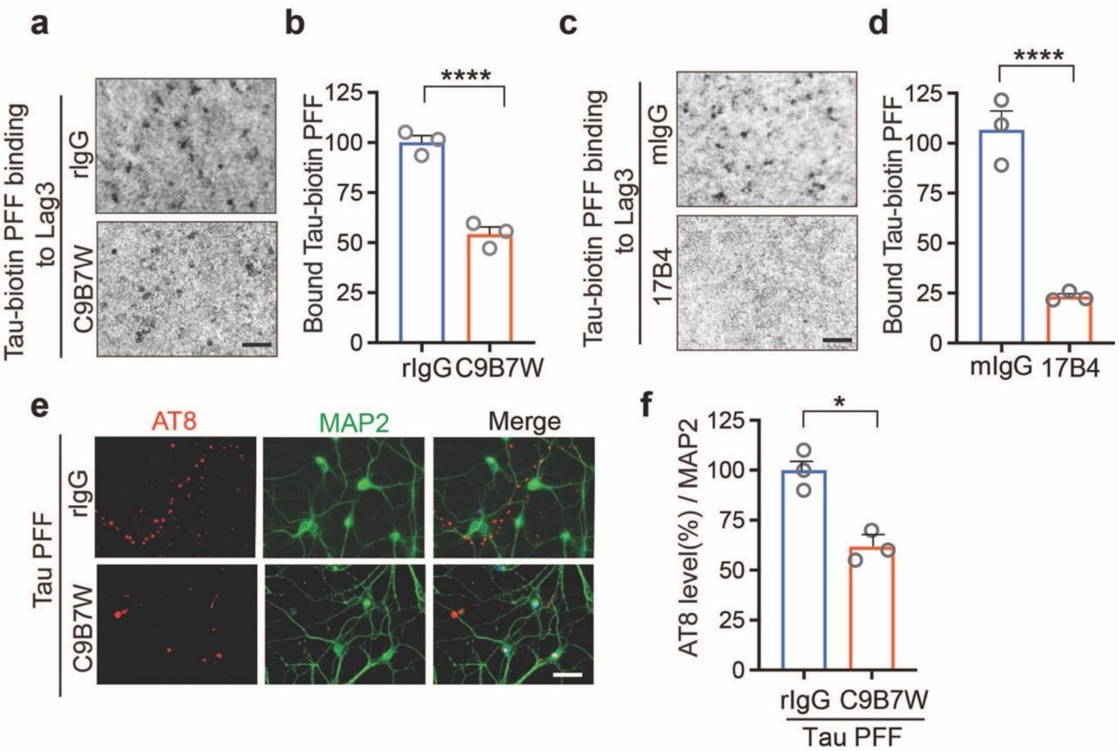

Figure S11: Lag3/LAG3 antibodies block Tau PFF binding to Lag3/LAG3, and subsequent pathologic propagation. │ **a.** Anti-Lag3 C9B7W blocks the binding of Tau-biotin PFF to Lag3-expressing SH-SY5Y cells. **b.** Quantification of a. Error bars represent means ± SEM, n = 3 independent experiments, Student's t-test. ****$P < 0.0001$. **c**. Anti-human-LAG3 antibody 17B4 blocks the binding of Tau-biotin PFF to human LAG3-expressing SH-SY5Y cells. **d.** Quantification of c, Error bars represents means ± SEM, n = 3 independent experiments, Student's t-test. ****$P < 0.0001$. **e.** AT8 phosphorylated Tau (P-Tau) was reduced by treatment with C9B7W in primary cortical neurons. **f.** Quantification of e. Error bars represent means ± SEM, n = 3 independent experiments, hyper-phosphorylated Tau at Ser202, Thr205 (AT8) levels were normalized with neuronal marker MAP2 signal. Student's t-test. *$P < 0.05$, Scale bar, 50 μm.

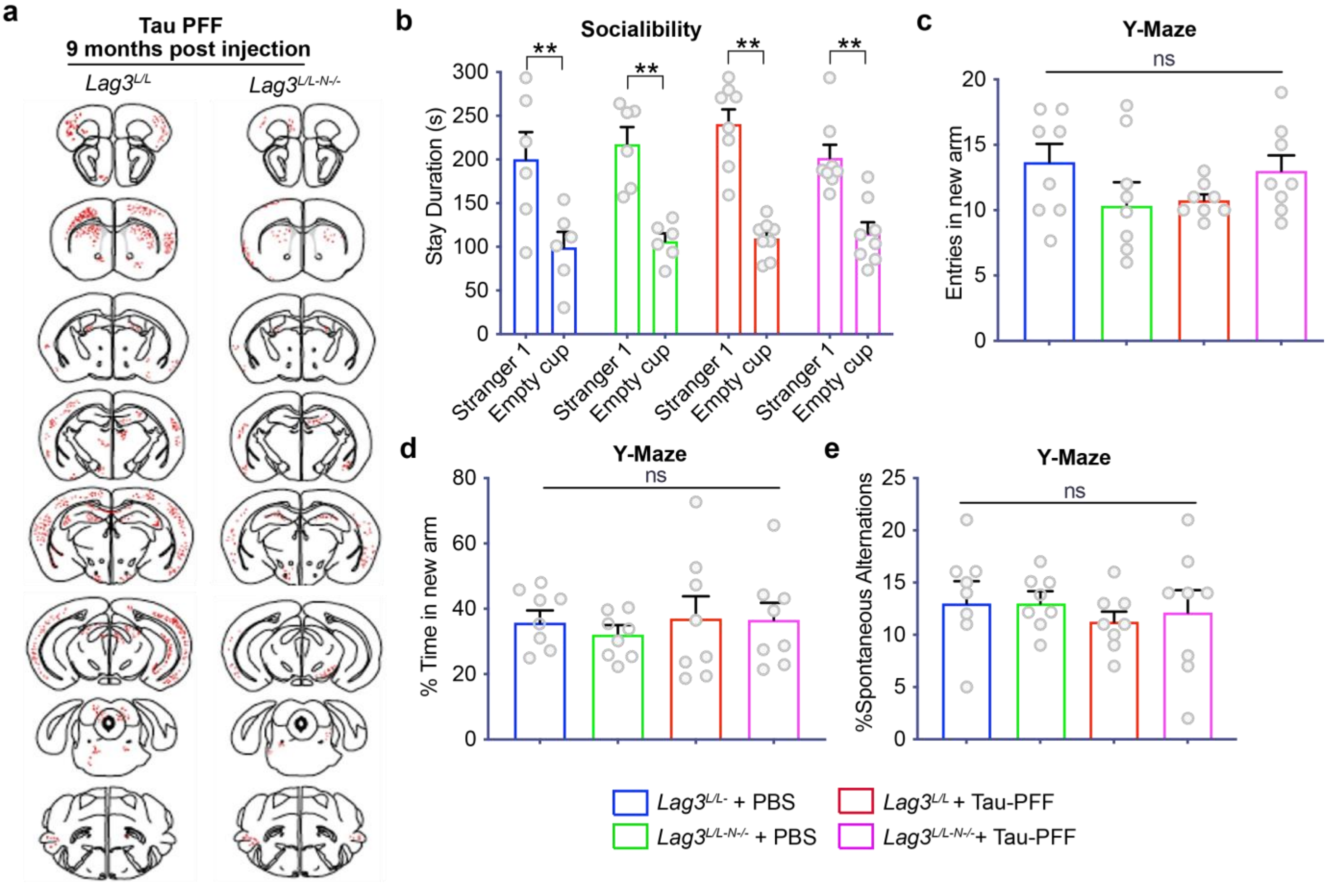

Figure S12: In vivo transmission and behavioral assessment of Tau PFF injected *Lag3*$^{L/L-N-/-}$ mice. │ **a.** Representative distribution of Tau pathology (red dots) on coronal sections stained for AT8 distribution of Tau PFF-injected mice**. b.**Total stay duration in the empty cup and stranger cup in three chamber social interaction test. Student's *t*-test. *$P < 0.01$, **c-e.** *Lag3*$^{L/L-N-/-}$ mice did not exhibit significant behavioral deficit in the Y-maze test. Specifically, number of entries in the new arm (**c**), percent time spent in the new arm (**d**), percentage of spontaneous alteration of behavior **(e),** Error bars represent SEM, $n = 8$ mice.
